# Cannabidiol triggers fatty acids β-oxidation mediated by Stat2 to facilitate intestinal stem cells regeneration post radiation

**DOI:** 10.1038/s12276-026-01711-5

**Published:** 2026-05-12

**Authors:** Zebin Liao, Congshu Huang, Liangliang Zhang, Changkun Hu, Zekun Wu, Zhijie Bai, Gaofu Li, Lei Zhou, Ningning Wang, Chaoji Huangfu, Zhexin Ni, Pan Shen, Wei Zhou, Yue Gao

**Affiliations:** 1 Academy of Military Medical Sciences, Beijing, China; 2 Traditional Chinese Medicine School, Henan University of Chinese Medicine, Zhenzhou, China; 3 Tianjin University of Traditional Chinese Medicine, Tianjin, China; 4 College of Pharmacy, Guangdong Pharmaceutical University, Guangzhou, China

**Keywords:** Intestinal stem cells, Lipid signalling

## Abstract

The development of compounds triggering intestinal stem cells (ISCs) proliferation represents a promising strategy to alleviate irradiation (IR)-induced gastrointestinal syndrome. Here, cannabidiol (CBD)-a nonpsychotomimetic phytocannabinoid derived from the *Cannabis sativa* plant-was found to dramatically improve body weight loss of mice and stimulate Lgr5^+^ ISCs proliferation upon a lethal dose of IR. Using absolute quantitative lipidomics, we found that the dysregulation of fatty acids in crypts induced by IR was rescued by CBD, which was indispensable for ISCs regeneration. Integrative analysis of transcriptome and lipidomics unveiled the critical role of PPARα in regulating fatty acid β-oxidation (FAO) by transcriptionally upregulating *Slc27a2* and *Acox1*. Further experiments showed that CBD could trigger the enrichment of Stat2 on the promoter region of *Pparα*, ultimately facilitating the FAO program and subsequent ISCs proliferation following IR exposure. In addition,THOC3 was identified as a direct target of CBD, which stabilized the THOC3 protein and substantially alleviated the IR-induced blockade of Stat2 mRNA nuclear export. This study reveals a connection between CBD-driven ISCs proliferation and the FAO program during IR damage, providing a promising avenue for IR-induced gastrointestinal syndrome treatment.

**Figure:**
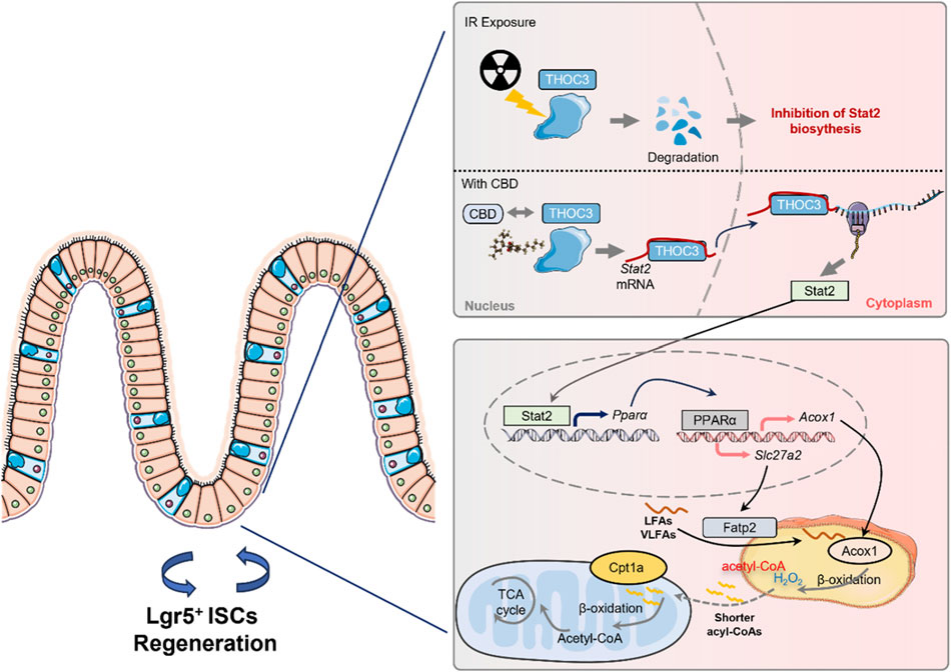
The binding of CBD to THOC3 maintains its radiation stability, which then supports the nuclear export of Stat2 mRNA for the subsequent transactivation of *Pparα.* The upregulation of PPARα will ultimately stimulate the FAO program, thereby facilitating ISCs regeneration during IR exposure.

The binding of CBD to THOC3 maintains its radiation stability, which then supports the nuclear export of Stat2 mRNA for the subsequent transactivation of *Pparα.* The upregulation of PPARα will ultimately stimulate the FAO program, thereby facilitating ISCs regeneration during IR exposure.

## CBD enhances fatty acid oxidation to support intestinal stem cells survival after radiation

The rapid self-renewal of the intestinal epithelium makes it vulnerable to high-dose irradiation, often leading to gastrointestinal syndrome in patients with cancer undergoing radiotherapy. This study explores cannabidiol (CBD) as a potential therapeutic agent to protect intestinal stem cells (ISCs) from radiation-induced damage. Researchers found that CBD markedly improved the survival and regeneration of Lgr5^+^ ISCs in irradiated mice. The study used various methods, including lipidomics and RNA sequencing, to demonstrate that CBD modulates fatty acid metabolism, enhancing ISCs proliferation. Key findings revealed that CBD stabilizes the THOC3 protein, facilitating the nuclear export of Stat2 mRNA, which in turn activates PPARα-mediated fatty acids oxidation. This process supports ISCs proliferation and intestinal regeneration. The study suggests that CBD could serve as a promising radioprotective agent, with future research needed to explore its broader applications in radiation therapy.

This summary was initially drafted using artificial intelligence, then revised and fact-checked by the author.

## Introduction

The rapid self-renewal of the intestinal epithelium renders it particularly vulnerable to high-dose irradiation (IR) from nuclear leak or intensive radiotherapy for abdominal and pelvic neoplasms treatment^1^. As the small intestine regenerates about once every 3 days, insufficient compensation for the extensive loss of tissues will inevitably cause gastrointestinal syndrome (GIS) characterized by diarrhea, bloating, bleeding, nausea, fecal urgency and even death^2^. Nowadays, approximately 50–60% of patients with cancer receive radiotherapy, but GIS induced by IR has confined the dose escalation for better curative effects^3^. Nevertheless, there is currently no US Food and Drug Administration (FDA)-approved countermeasures for IR-induced GIS and the unmet medical needs are unmet. Intestinal stem cells (ISCs) expressing leucine-rich repeat-containing G-protein coupled receptor 5 (Lgr5) are the primary source for maintaining the crypt–villus structure^4^. Extensive research has demonstrated the critical role of ISCs in functional intestinal cell differentiation to maintain physiological homeostasis or the continual regeneration of intestinal epithelium^5,6^. As the number of Lgr5^+^ ISCs decreases rapidly and dramatically upon IR exposure, the development of drugs protecting these cells could be a potential therapeutic avenue for IR-induced GIS.

The processes involved in the metabolic program of fatty acids (FAs) that are secreted upon the lipolysis of neutral lipids has been demonstrated to favor ISCs maintenance and function^7^. FAs can be metabolized by β-oxidation in peroxisomes or mitochondria to produce substrates for energy metabolism and acetyl-CoA for chromatin remodeling, maintaining the pluripotency and proliferation of ISCs^8–10^. For instance, short-term fasting or stimulation of the peroxisome proliferator-activated receptor (PPAR) family, hepatocyte nuclear factor 4 (HNF4) or PR-domain containing 16 (PRDM16) ultimately activate the FAs β-oxidation (FAO) program or genes, thereby increasing the number of ISCs and enhancing their self-renewal potentiality^11–14^. Furthermore, genetic ablation of the rate-limiting enzyme in FAO (Cpt1a) or inhibition of PPARα with cholic acid impeded FAO and markedly decreased ISCs numbers and function^11,15^. Correspondingly, supplementation with FAO substrates, such as short-chain FAs (propionic acid and butyric acid) derived from microbial groups, or FAO products (β-hydroxybutyric acid or acetic acid) sustained and improved ISCs function^16,17^. By contrast, disruption of the Arf1-mediated lipolysis pathway or the acetyl-CoA carboxylase 1 (ACC1)-mediated de novo fatty acid synthesis pathway resulted in a marked decline in ISCs^7,18^. However, current studies are only beginning to determine the crucial role of fatty acid metabolism in ISCs, and the detailed mechanisms regulating fatty acids mobilization, catabolism, biosynthesis and transport in response to external stimuli remain largely unclear. Furthermore, no therapeutic agents or strategies have been reported to promote ISCs-driven intestinal regeneration by targeting fatty acid metabolism or mobilization following IR exposure.

Cannabidiol (CBD) is a nonpsychotomimetic phytocannabinoid derived from the *Cannabis sativa* plant, which possesses many therapeutic properties. With very low toxicity (the LD_50_ is 212 mg/kg for rhesus monkeys^19^) CBD alone (Epidiolex) or in combination with tetrahydrocannabinol (THC) (Sativex/Nabiximols) have been licensed for the treatment of seizures associated with Lennox–Gastaut syndrome, Dravet syndrome and tuberous sclerosis^20^ and spasticity in moderate to severe multiple sclerosis^21^, respectively. CBD is also a promising compound for gastrointestinal tract diseases, of which refractory chemotherapy-induced nausea and vomiting^22^, intestinal bowel diseases^23^ and permeability in the human colon^24^ could be markedly eased with CBD treatment. In recent years, CBD has been demonstrated to modulate the proliferation, migration, metabolism or differentiation of stem cells. For instance, CBD treatment has been shown to confine the overactivation of radial neural stem cells, thereby maintaining normal neurogenesiss^25^; attenuate endoplasmic reticulum stress, thus protecting oligodendrocyte progenitor cells from inflammation-induced apoptosis^26^; enhance the viability and proliferation of skeletal stem/progenitor cells, thereby preventing osteoporosis^27^ and so on. Several studies have shown that CBD can remodel lipid metabolism. For example, CBD inhibits phospholipid peroxidation induced by UV IR^28,29^, lipogenic activity in sebocytes and muscle^30,31^ and lipid accumulation-mediated hepatosteatosis, while promoting cholesterol metabolism-related gene expression in microglial cells^32^ and adipogenesis in mesenchymal stem cells^33^. We hypothesized that CBD may serve as a potential agent to facilitate ISCs proliferation by modulating fatty acids metabolism following IR-induced damage.

In the present study, IR-induced depletion of Lgr5⁺ ISCs was markedly rescued by CBD treatment. Mechanistically, CBD directly bound to THO complex subunit 3 (THOC3), stabilizing the protein upon IR exposure and promoting the nuclear export of Stat2 mRNA to enable its translation. Stat2 then acted as a transcription factor to upregulate PPARα, which subsequently transactivated genes involved in fatty acids transport and β-oxidation, including *Slc27a2* and *Acox1*. This CBD-mediated remodeling of fatty acids metabolism ultimately supported ISCs proliferation and intestinal regeneration following IR injury.

## Materials and methods

### Animals and IR treatment

**Fig. 1:**
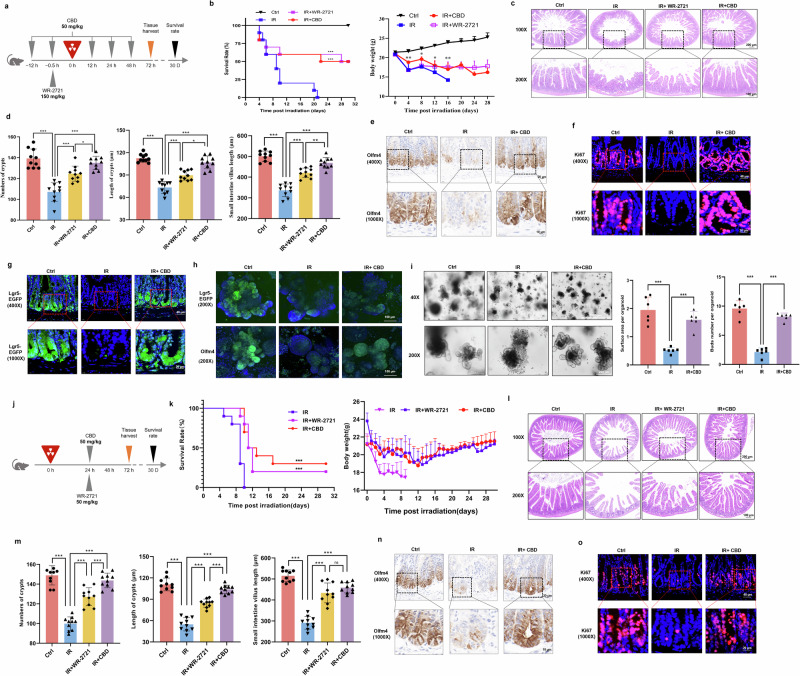
The radioprotective effect of CBD against IR-induced intestinal injury. **a** A schematic overview of experiment in which the mice were treated with agents before IR. **b**–**f**, CBD treatment profoundly improved the survival rate and body weight loss of mice exposed to a lethal dosage of IR (**b**) and alleviated IR-induced damage on the intestinal structure (**c** and **d**), Olfm4^+^ ISCs (**e**) and Ki67^+^ transit-amplifying (TA) cells (**f**). **g** Proliferation of Lgr5^+^ ISCs were determined with Lgr5-EGFP-IRES-CreERT2 mice. **h** Intestinal organoids were culture and Lgr5^+^/Olfm4^+^ ISCs were detected with IF staining. **i** Representative images and quantitation of intestinal organoids. **j** A schematic overview of the experiment in which mice were treated with agents post IR. **k**–**o**, The survival rate and body weight loss of mice (**k**), damage to the intestinal structure (**l** and **m**) and the change of Olfm4^+^ ISCs (**n**) and Ki67^+^ TA cells (**o**) were determined. The results are expressed as the mean ± s.d. *P < 0.05, **P < 0.01 and ***P < 0.001. ns, not significant. Ctrl, control.

All animal experiments were approved by Ethics Committee of Animal Experiments of Academy of Military Medical Sciences (IACUC-DWZX-2021-557). Male C57BL/6J mice (20–22 g) were obtained from Beijing Vital River Laboratory Animal Technology. Lgr5-EGFP-IRES-creERT2 mice were purchased from Cyagen Biosciences. The appropriate environment with a standard 12-h light/12-h dark cycle was provided, and water and food could be obtained ad libitum. CBD (50 mg/kg, Yunnan Hemp Biotechnology) dissolved in soybean oil and WR-2721 (150 mg/kg, MedChemExpress) dissolved in normal saline were used for intraperitoneal injection according to the scheme in Fig. 1a. Mice (*n* = 10) received total body IR at a rate of 0.6 Gy/min with a ^60^Co irradiator (8.5 Gy, Academy of Military Medical Sciences). Small intestines were collected 3 days post IR for histological analysis.

### Intestinal organoids culture and treatments

Small intestines were cut into ~2–3-mm^2^ fragments and then incubated with PBS containing 2 mM EDTA at 4 ℃ for 40 min. After vibration with a vortex mixer for 2 min, the enriched crypts were obtained by filtering the suspension through a 70-μm filter. Crypts were resuspended with IntestiCult Organoid Growth Medium (Stemcell Technologies) and then mixed with Matrigel (Corning) in the plates. Before IR (8 Gy, 0.6 Gy/min), organoids were treated with CBD (10 μM), GW6471 (MedChemExpress, 4 μM), GW590735 (MedChemExpress, 1 nM), lipofermata (MedChemExpress, 10 μM), 10,12-tricosadiynoic acid (MedChemExpress, 1 μM), γ-linolenic acid (MedChemExpress, 2 μM), α-linolenic acid (MedChemExpress, 2 μM), eicosapentaenoic acid (EPA) (MedChemExpress, 2 μM) and *cis*-9-palmitoleic acid (Sigma-Aldrich, 3 μM). The medium was replaced every 3 days.

### Histological analysis

Small intestines were fixed in 10% formalin for 24 h and then processed by dehydration and paraffin embedding. The samples were sectioned into 3-μm slices for hematoxylin and eosin (H&E) staining, tunel staining and immunohistochemical or immunofluorescent (IF) assays. Target retrieval solution (DAKO) was used to retrieve the antigen and then tissues were incubated with antibodies against Olfm4 (1:800, CST, 39141), PPARα (1:500, Proteintech, 66826-1-Ig) and Stat2 (1:300, CST, 72604). Proteins levels were detected with biotin-conjugated anti-rabbit IgG for immunocytochemistry. For IF in situ, antibodies against Ki67 (1:400, CST, 9129), EGFP (1:200, Abcam, ab184601), γH2AX (1:250, Abcam, ab81299), lysozyme (1:250, Abcam, ab108508), SOX9 (1:500, CST, 82630), Fatp2 (1:500, Proteintech, 14048-1-AP), Acox1 (1:250, Abcam, ab184032) and THOC3 (1:250, Santa Cruz, sc-377456) were used and the signal was detected with secondary antibodies (Alexa-Fluor-488 and Alexa-Fluor-594).

### Laser confocal fluorescence microscopy

Intestinal organoids were first fixed with 4% formalin for 90 min and permeated by 0.5% Triton X-100 for 30 min. After treatment with 2% BSA, organoids were incubated with antibodies against EGFP (1:200, Abcam, ab184601), Olfm4 (1:200, CST, 39141), PPARα (1:200, Proteintech, 66826-1-Ig), Stat2 (1:200, CST, 72604), p-Stat2 (Tyr690) (1:200, Thermo Fisher Scientific, 600-401-A93), Fatp2 (1:200, Proteintech, 14048-1-AP), Acox1 (1:200, Abcam, ab184032) or THOC3 (1:200, Santa Cruz, sc-377456) at 4 °C overnight. Goat anti-rabbit IgG Alexa Fluro 488 or 647 conjugate (CST) was used as a secondary antibody, and after incubation with 4′,6-diamidino-2-phenylindole (DAPI; Sigma-Aldrich), the organoids were scanned and their three-dimensionally reconstructed with laser confocal fluorescence microscopy (Zeiss).

### Lipidomics analysis

The absolute quantitative lipidomics were supported by Shanghai Applied Protein Technology. Briefly, intestinal crypts isolated from mice treated or untreated with CBD upon IR exposure were spiked with 200 μl of methanol, 20 μl of internal lipid standards and 800 μl of MTBE. At 30 min post ultrasound treatment, 200 μl of water was added to the mixture. The organic phase was obtained and dried under nitrogen after the solution was vortexed and centrifuged (14,000 rpm, 4 °C, 15 min). The samples were separated in an ultrahigh performance liquid chromatography system (UHPLC Nexera LC-30A) with a C18 and HILIC NH_2_ chromatographic column and then subjected to mass spectrometry with an AB 6500+ QTRAP (AB SCIEX). The R package MetaboAnalystR (v4.0.0) was used to normalize the abundance of lipids using quantity control samples. An orthogonal partial least squares-discriminant analysis was used to identify differentially abundant lipids (DALs). Lipids with a variable importance in projection value >1 and a significance level at a *P* value <0.05 were considered as statistically significant lipids in abundance.

### GC–MS analysis

Intestinal crypts were resuspended with 20% phosphoric acid and 500 μM 4-methylvaleric acid was used as internal standard. After centrifuging at 14,000*g*, 1 μl sample was injected and separated with gas chromatography (GC) system (carrier gas was helium, carrier gas flow rate was 1.0 ml/min) equipped with an Agilent DB-FFAP capillary column (30 mm × 250 μm × 0.25 mm). A 5977BMSD mass spectrometer (Agilent) was used for mass spectrometry (MS) analysis (inlet temperature 250 °C, ion source temperature 230 °C, transmission line temperature 250 °C, quadrupole temperature 150 °C). The energy of the electron bombardment ionization source was 70 eV and the SCAN/SIM mode was adopted for detection.

### RNA sequencing and analysis

Intestinal crypts were treated with TRIzol (Invitrogen, Thermo Fisher Scientific) to extract RNA for sequencing. After the completion of library preparation, paired-end sequencing was performed on the Illumina NovaSeq X Plus platform. Clean reads were obtained using fastp (v0.23.2) to trim adapters and low-quality reads. Then, reads were aligned to the mouse reference genome mm10 using Salmon (v1.10.2) to quantify transcript-level expression. The R package tximport (v1.26.1) was utilized to convert transcript-level expression to gene-level expression in each sample. The R package DESeq2 (v1.40.2) was used to perform differential expression analysis, calculating fold change (FC) and *P* value using the Wald test. Differentially expressed genes (DEGs) were identified as genes with absolute FC >1.5 and adjusted *P* values <0.05 by the Benjamini–Hochberg method.

### Quantitative real-time PCR

TRIzol reagent (Invitrogen, Thermo Fisher Scientific) was used to extract total RNA. For the extraction of nuclear and cytoplasmic RNA, the Nuclear Extraction kit (Solarbio Life Sciences) was used. cDNA was synthesized with reverse transcription, and real-time PCR was performance with the SYBR Green Realtime PCR Master Mix (Vazyme Biotech) on an Applied Biosystems (StepOnePlus 272006169). The primer sequences are included in Supplementary Table 1.

### Western blot assay

Mammalian protein extraction reagent (Thermo Fisher Scientific) was used to extract total proteins in crypts or organoids. After determining the protein concentrations with the BCA protein assay kit (Thermo Fisher Scientific), equal amounts of protein were subjected to SDS–polyacrylamide gel electrophoresis and transferred to a PVDF membrane (Millipore). The membranes were blocked with 5% skim milk and then incubated overnight at 4 °C with primary antibodies against PPARα (1:2,000, Proteintech, 66826-1-Ig), Stat2 (1:2,000, CST, 72604), p-Stat2 (Tyr690) (1:1,000, Thermo Fisher Scientific, 600-401-A93), Fatp2 (1:2,000, Proteintech, 14048-1-AP), Acox1 (1:2,000, Abcam, ab184032) or THOC3 (1:2,000, Santa Cruz, sc-377456). Blots were then incubated with a secondary antibody at room temperature for 1 h and GAPDH was used to normalize the expression levels of the total proteins.

### Lentivirus infection

Lentivirus were constructed by Hanheng Science and Technology. The specific short hairpin RNA oligonucleotide sequences targeting mouse *Stat2* and *THOC3* are listed in Supplementary Table 2. To overexpress *Slc27a2*, *Stat2* or *THOC3*, mouse full-length Slc27a2, Stat2 or THOC3 expression vectors were generated by PCR-based amplification of cDNA. Intestinal organoids were first dissociated by Gentle Cell Dissociation Reagent (GCDR, Stemcell Technologies) and cold DMEM/F12 was used to resuspended the fragments. Lentivirus were added to the suspensions for 6 h (37°C, 5% CO_2_) and then seeded onto Matrigel as previously described.

### FAO measurement

The Mitochondrial Extraction kit (Solarbio Life Sciences) was used to collected adequate mitochondria from intestinal crypts for FAO measurement. The FAO rate colorimetric assay kit (Genmed Scientifics, GMS50679) was then used according to manufacturer’s instructions. Briefly, the mitochondria were resuspended and the reduction rate of ferricyanide triggered by electrons generated from the oxidation of palmitoyl carnitine was detected. The FAO rate was evaluated by calculating the corresponding relationship between the absorption value (420 nm) and time.

### Mitochondrial membrane potential measurement

The enhanced mitochondrial membrane potential assay kit with JC-1 purchased from Beyotime Biotechnology was used according to the manufacturer’s instructions. Intestinal organoids were seeded in a confocal dish and washed with cold PBS to eliminate Matrigel. JC-1 (200×) was diluted with JC-1 staining buffer solution to obtain the JC-1 working buffer. Then 0.5 ml of JC-1 working buffer was added to each dish and incubated at 37 °C for 20 min. JC-1 monomers and aggregates were visualized with a laser scanning confocal microscope at 490 nm and 530 nm, respectively.

### ChIP assay

For chromatin immunoprecipitation (ChIP), the transcription factor-DNA binding interactions were identified with the Pierce Agarose ChIP kit (26156, Thermo Fisher Scientific). Intestinal crypts were isolated and crosslinked with 1% formaldehyde, then the pellets were processed to neutralization with glycine solution. After micrococcal nuclease digestion, the digested chromatin was subjected to immunoprecipitation (IP), in which normal rabbit IgG was used as negative control. The IP product was eluted and incubated with 5 M NaCl and 20 mg/ml Proteinase K, and then DNA was recovered with a DNA binding buffer and DNA column wash buffer. The purified DNA was obtained and used for qPCR detection. The primers of the promoter region are listed in supplementary Supplementary Table 3.

### Dual-luciferase reporter assay

Plasmids containing sequences of transcription factors or promoter (Supplementary Table 4**)** were constructed by Shanghai Zorin Biological Technology. First, 293T cells were inoculated in a 24-well plate to an appropriate number and a mixed solution of plasmid and X-tremegene HP transfection reagent was added. After 48 h, the Dual-Luciferase Reporter Assay System kit (E1910, Promega) was used to treat cells and detect the luciferase signal, in which firefly luminescence was first detected by a microplate analyzer. Then, Stop & Glo reagent was added, and Renilla luminescence was detected.

### Pull down of CBD-binding proteins

As streptavidin can strongly bind to biotin, streptavidin agarose beads (Millipore, S1638) and biotinylated CBD were used. First, 500 μl beads were washed with cold PBS three times, then 500 μl PBS containing biotinylated CBD (10 μM) or an equivalent volume of biotin was added to the beads. After incubating overnight at 4 °C, the beads were collected with centrifugation (14,000*g*, 5 s) and washed with cold PBS three times. Total proteins from intestinal crypts were extracted, which were then incubated with the beads, and 100 μM CBD was used in the competitive binding group. After incubating overnight at 4 °C, the beads were collected with centrifugation (14,000*g*, 5 s) and washed with cold PBS three times.

### Label-free proteomics analysis

The streptavidin agarose beads collected above were processed for label-free proteomics analysis, which was supported by Beijing Qinglian Biotech Briefly, beads were washed with PBS containing reductive alkylation reagent and the eluent was subjected to digestion. An Orbitrap Eclipse Mass Spectrometer equiped with AIMS Pro Interface was used, and the full scanning range of MS was *m*/*z* 350–2,000. The resolution of primary MS was set to 12,0000 (200 *m*/*z*), and the secondary MS was 15,000 (200 *m*/*z*). The raw files were searched with Proteome Discoverer2.4 and the filtered expression matrix underwent variance stabilizing transformation using the R package vsn (v3.68.0). Differential protein expression analysis was performed using the R package DEP (version 1.22.0), which applies protein-wise linear models and empirical Bayes statistics based on the R package limma (v3.56.2). False discovery rates (FDRs) were estimated using the R package fdrtool (v1.2.17). Proteins with an absolute FC greater than 2 and FDR <0.05 were considered as differentially expressed proteins (DEPs).

### Molecular docking

The molecular structure of CBD was obtained from PubChem (compound CID: 644019) and the protein structures of THOC3, Rnasel, Zwilch, Zfyve26 and COMMD1 were downloaded from the PDB database. The above structures were aligned into pdb format, and were then subjected to docking with AutoDock Vina. PyMOL (2.2.0) was used for visualizing the docking results.

### CETSA

For the cellular thermal shift assay (CETSA), intestinal crypts were treated with five freeze–thaw cycles by liquid nitrogen and the lysates were subsequently mixed with CBD (10 μM) or an equal volume of solvent for 1 h at room temperature. Then the lysate was incubated at incremental temperature (46 °C, 49 °C, 52 °C, 55 °C, 58 °C, 61 °C, 64 °C and 67 °C) for 3 min. After cooling at room temperature for 5 min, the lysate was mixed with loading buffer and used for the western blot assay.

### DARTS

For the drug affinity-responsive target stability (DARTS) assay, crypt lysates collected as above were treated with different concentrations of CBD (5 μM, 10 μM, 20 μM or 40 μM) or left untreated for 1 h, then 10 μg/ml pronase was added to lysates at room temperature for 15 min. The protein levels were analyzed by a western blot assay.

### SPR

For surface plasmon resonance (SPR), mouse THOC3 recombinant protein was purchased form EIAab Technology (R16108m), which was attached to a Sensor chip NTA (Biacore, GE Healthcare). Five concentrations of CBD were injected at the association phase (25 °C), and experimental data were collected and analyzed to fit an appropriate binding model to obtain the equilibrium dissociation constant (*K*_d_).

### RNAscope assay

Fluorescence in situ hybridization for *Lgr5* was performed with RNAscope Multiplex Fluorescent Reagent kit v2 (ACD) and the mRNA probe was also synthesized by ACD (RNAscope Probe-Mm-Lgr5). Small intestine slides were baking at 60 °C and thawed at room temperature for 10 min and then post-fixed in prechilled 4% PFA and washed with PBS. After dehydration through 50%, 70%, 100% and 100% ethanol, the slides were air dried and loaded onto a Bond Rx instrument (Leica Biosystems). The slides were subsequently treated with Epitope Retrieval Solution 2 (Leica Biosystems) and ACD Enzyme from the Multiplex Reagent kit in turn. After probe hybridization and signal amplification, TSA plus fluorphores were used to detect RNAscope probe. To determine the localization of THOC3 and Stat2, the slides prepared above were processed for the IF assay.

### RNA immunoprecipitation

An RNA Immunoprecipitation kit (Bes5101, BersinBio) was used according to the manufacturer’s instruction. In short, lysis buffer containing protease inhibitors and RNA enzyme inhibitors were added to intestinal crypts isolated from mice treated with CBD or untreated, which were then subjected for the DNA removal process. Antibodies against THOC3 (Santa Cruz, sc-377456) and IgG (Bes5101, BersinBio) were incubated with cell lysates at 4 °C overnight, then the RNA–protein complexes were isolated. After Proteinase K digestion, protein-bound RNAs were extracted with TRIzol, and RT–qPCR was performed to analyze the expression levels of RNA.

### RNA pulldown assay

The RNA pulldown kit (Bes5102, BersinBio) was used according to the manufacturer’s instruction. The 5′-biotin-labeled Stat2 and LacZ probes were synthesized by BersinBio. Intestinal crypts from mice treated with CBD or untreated for 12 h were isolated to extract whole proteins and the nucleic acid from protein sample was removed to obtain cell lysates. Then, the RNA probe was incubated with streptavidin magnetic beads for 30 min and cell lysates were incubated with the beads coated with RNA probe for 2 h at room temperature. The RNA-binding proteins were then eluted for the western blot assay.

### Quantification and statistical analysis

Data are presented as mean and s.d. and the statistical analysis was performed using GraphPad Prism 7.0. Single comparisons were made using either paired or unpaired Student’s *t*-tests where appropriate, as indicated in the figure legends.

## Results

### CBD favors Lgr5^+^ ISCs proliferation and subsequent intestinal regeneration after IR damage

To investigate the radioprotective activity of CBD against intestinal injury, mice was exposed to lethal-dose IR and the small intestine was subjected for further examination (Fig. 1a). We observed that CBD treatment could profoundly improve the body weight loss and survival rate of mice, alleviate the destruction on the intestinal structure and inhibit cell apoptotic and DNA damage on intestinal crypts upon IR exposure. The radioprotective effect of CBD was superior to that of amifostine (WR-2721) (Fig. 1b–d and Supplementary Fig. 1a, b). In parallel, an exaggerated inflammatory response in intestinal crypts induced by IR damage was strikingly inhibited by CBD (Supplementary Fig. 2). From IF and immunohistochemistry assays, the damage on ISCs labeled with Olfm4 and proliferative cells labeled with Ki67 induced by IR were found to be eased by CBD (Fig. 1e, f), while there was no significant change on lysozyme^+^ Paneth cells, SOX9^+^ ISCs, MUC2^+^ goblet cells and ChgA^+^ endocrine cells (Supplementary Fig. 1c–g). Since CBD has no significant effect on the proliferation of differentiated cells such as Paneth cells, goblet cells and endocrine cells, we hypothesize that it specifically promotes the proliferation of ISCs upon IR damage. Thus, Lgr5-EGFP-IRES-CreERT2 mice were used to trace the proliferation and regeneration of Lgr5^+^ ISCs, wherein impaired Lgr5-EGFP signal during IR exposure rescued by CBD in intestinal crypts and intestinal organoids was observed (Fig. 1g–i). To confirm whether CBD could promote ISCs proliferation after injury, we subsequently administered CBD at 24 h post IR (Fig. 1j). The results showed that the body weight loss, decrease in survival rate, intestinal structure destruction and ISCs depletion could be markedly eased by CBD (Fig. 1k–o) and the radioprotective effect of CBD was superior to that of WR-2721 (Fig. 1k–m). Nevertheless, there was no obvious difference between CBD and WR-2721 in their effects on tissue regeneration at 14 days post-IR injury (Supplementary Fig. 1h–j). The above results demonstrated that CBD serves as a potent mitigator, alleviating IR-induced GIS by facilitating Lgr5^+^ ISCs regeneration and survival.

### CBD rescues FAs dysregulation from IR damage in intestinal crypts

As CBD has been reported to modulate lipid metabolism, lipidomics sequencing was performed to gain insights into whether lipid metabolism was involved in the radioprotective effect of CBD on ISCs. Lipid metabolic disturbance on intestinal crypts was observed upon IR damage, in which 98 lipids were upregulated and 127 lipids downregulated, the main DALs included phosphatidylethanolamines (PEs), phosphatidylcholines (PCs) and triacylglycerols (TGs). As compared with the IR-damaged group, the proportion of DALs was dramatically altered by CBD treatment, with 51 lipids upregulated and 38 lipids downregulated, which suggests that lipid metabolic disturbance induced by IR damage was remodeled upon CBD treatment (Supplementary Fig. 3).

**Fig. 2:**
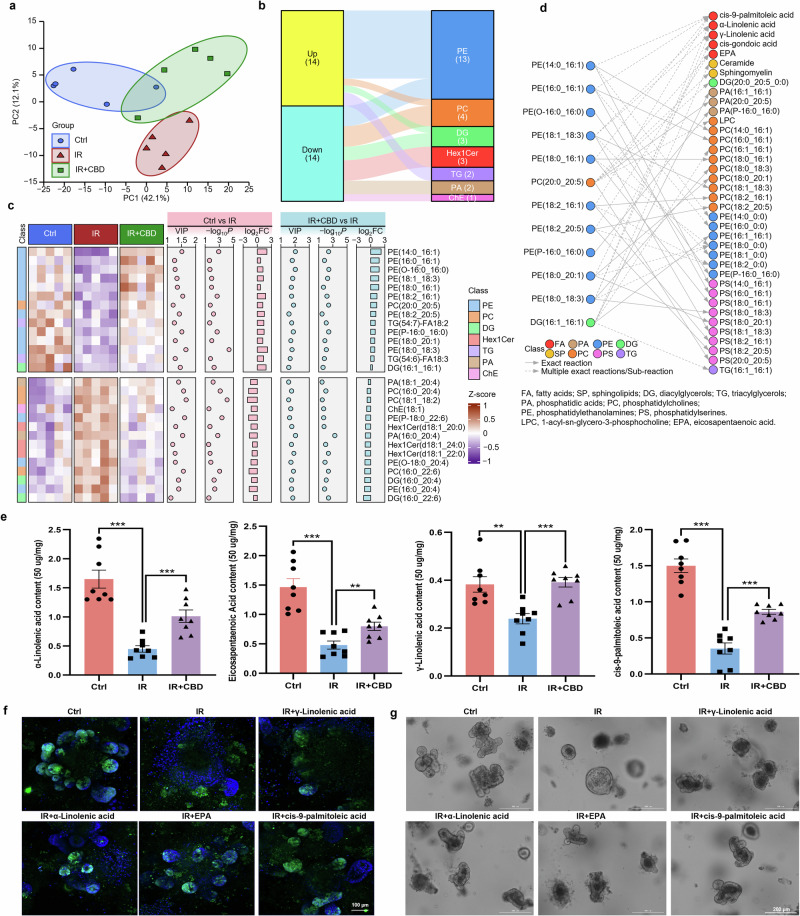
FAs dysregulation induced by IR damage was rescued by CBD. **a** Principal component analysis (PCA) results of the lipidomics in the Ctrl, IR and IR + CBD groups. **b** The class of DALs is shown in an alluvial plot. **c** The heat map displays the abundance change of 28 DALs and the left block represents the class of DALs, with scatter plots and bar charts on the right. **d** The biochemical reactions of DALs obtained from the LMSD. **e** The levels of FAs including γ-linolenic acid, α-linolenic acid, *cis*-9-palmitoleic acid and EPA detected by GC–MS. **f**, **g** The effects of exogenous FAs on Lgr5^+^ ISCs proliferation (**f**) and organoid buddings/surface (**g**) post IR. The results are expressed as the mean ± s.d. **P < 0.01 and ***P < 0.001.

The decreased levels of 14 lipids dysregulated by IR could be significantly restored with the pretreatment of CBD, in which ten of them belong to PEs (including PE(14:0_16:1), PE(16:0_16:1), PE(O-16:0_16:0), PE(18:1_18:3), PE(18:0_16:1), PE(18:2_16:1), PE(18:2_20:5), PE(P-16:0_16:0), PE(18:0_20:1) and PE(18:0_18:3)) (Fig. 2a–c). The biochemical reactions of these PEs were then analyzed with the LIPID MAPS Structure Database (LMSD), which is the largest public lipid-only database encompassing the structures and annotations of biologically relevant lipids^34^, in which these PEs can be metabolized into FAs including γ-linolenic acid, α-linolenic acid, *cis*-9-palmitoleic acid and EPA (Fig. 2d). Consistent with this, GC–MS showed that CBD could reverse the decreased levels of the above FAs in crypt cells during IR injury (Fig. 2e). To further validate the radioprotective effects of FAs delineated above, intestinal organoids derived from Lgr5-EGFP-IRES-CreERT2 mice were supplemented with exogenous FAs, and more budding rates and abundance signal of Lgr5-EGFP in FAs-treated organoids were observed as compared with the IR damage group (Fig. 2f,g and Supplementary Fig. 4a), implying the metabolism of FAs was involved in the proliferation and survival of ISCs stimulated by CBD upon IR exposure.

### FAO was identified to facilitate ISCs proliferation upon IR toxicity

**Fig. 3:**
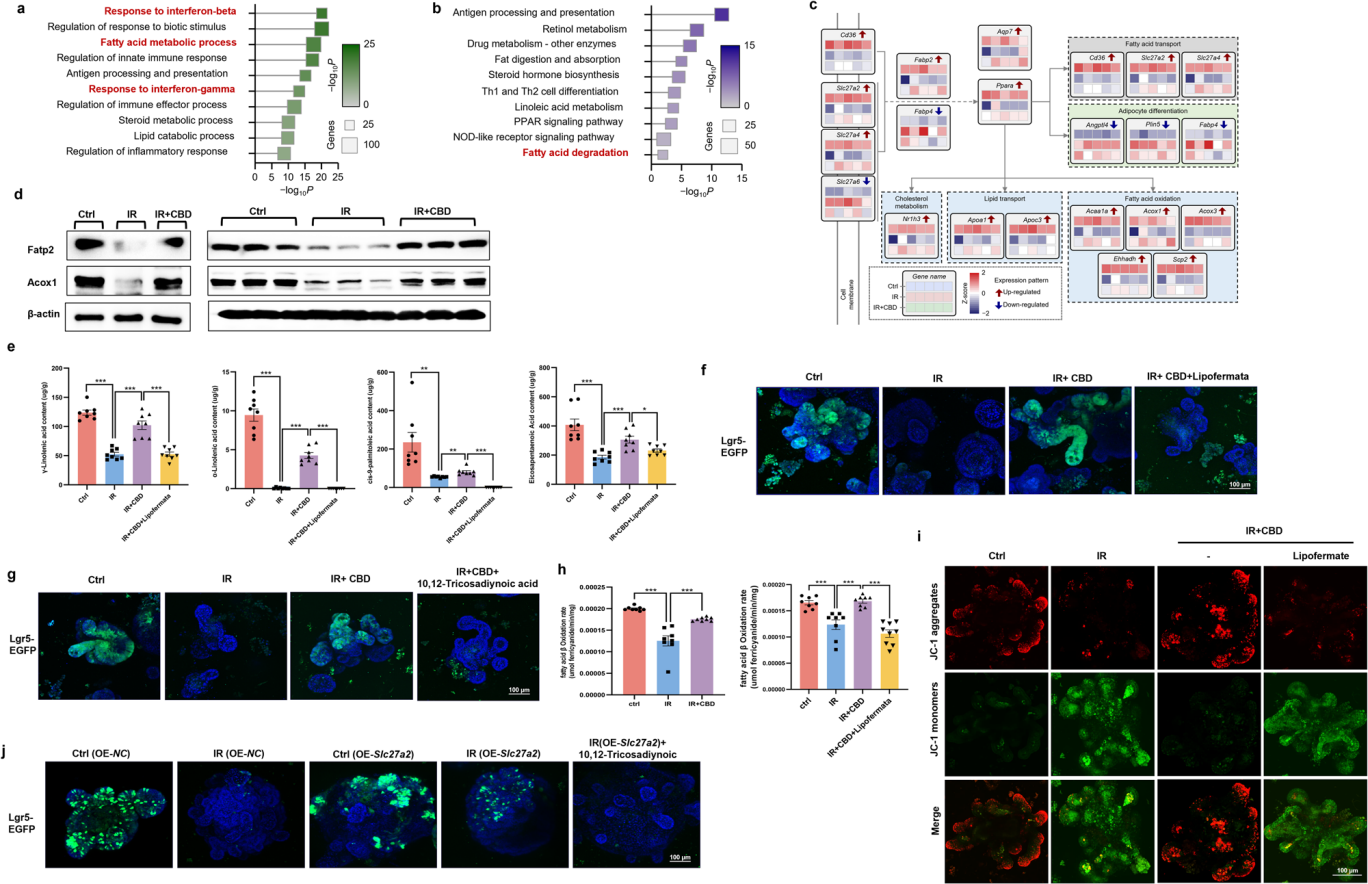
mRNA sequencing identified the essential role of Fatp2 and Acox1 in mediating ISCs regeneration upon IR exposure. **a**, **b** Enrichment results of biological processes (**a**) and KEGG pathways (**b**) based on the DEGs. Darker colors indicate higher enrichment levels and larger squares indicate a greater number of DEGs in that term. **c** The classification of DEGs involved in lipid metabolism. Each square represents a gene, and the heat maps, from top to bottom, represent the expression levels. **d** Protein levels of Fatp2 and Acox1 were detected with a western blot assay. **e** The levels of FAs including γ-linolenic acid, α-linolenic acid, *cis*-9-palmitoleic acid and EPA detected by GC–MS. **f,** **g** The radioprotective effect of CBD on ISCs (Lgr5^+^) was counteracted by Fatp2 inhibitor lipofermata (**f**) or Acox1 inhibitor 10,12-Tricosadiynoic acid (**g**). **h**, **i** The FAO rates of intestinal crypts (**h**) and mitochondrial membrane potential of intestinal organoids (**i**) were detected. **j** The overexpression of *Slc27a2* in organoids alleviated ISCs loss, while 10,12-tricosadiynoic acid treatment abolished this effect. The results are expressed as the mean ± s.d. *P < 0.05, **P < 0.01 and ***P< 0.001. OE overexpressing.

The above findings suggested that CBD modulated FAs metabolism, thus sustaining ISCs proliferation after IR injury, but the critical mechanism remained to be uncovered. Gene Ontology (GO) and Kyoto Encyclopedia of Genes and Genomes (KEGG) enrichment analysis of transcriptome data from mRNA sequencing showed that FA metabolic process and FA degradation pathways were activated by CBD after IR (Fig. 3a,b). DEGs related to lipid metabolism were selected for further study, and genes mediating FAO, that is, the process by which FAs are degraded (*Acaa1a, Acox1*, *Acox3*, *Ehhadh* and *Scp2*) and FA transport (*Slc27a2*, *CD36* and *Slc27a4*) were found to be stimulated by CBD (Fig. 3c and Supplementary Fig. 4b). As all of the above genes associated with FAO modulate the peroxisomal β-oxidation process, acyl-CoA oxidase 1 (Acox1), that is, the first rate-limiting enzyme initiating peroxisomal FAO, and *Slc27a2*, encoding FA transport protein 2 (Fatp2) localizing in peroxisome^35,36^, were selected for further investigation. Western blot assay showed that the expression of Fatp2 and Acox1 proteins in intestinal crypts or organoids were downregulated upon IR exposure, and CBD treatment could dramatically restore the levels of these proteins (Fig. 3d). Fatp2 and Acox1 were then stained by in situ IF and the results were observed (Supplementary Fig. 5a–c).

As idemonstrated above, the decreased FA levels of γ-linolenic acid, α-linolenic acid, *cis*-9-palmitoleic acid and EPA in crypts exposed to IR could be rescued by CBD, so the key role of FAs transport mediated by Fatp2 on the radioprotective effect of CBD was first investigated. It was found that the pharmacological inhibition of Fatp2 with lipofermata^37^ eliminated the activity of CBD to restore FAs levels, to favor the budding of intestinal organoids and to trigger the proliferation of Lgr5^+^ ISCs after IR damage (Fig. 3e,f and Supplementary Fig. 5e,f), while the overexpression of *Slc27a2* with lentivirus alleviated ISCs loss (Fig. 3j and Supplementary Fig. 5d,g). FAs serve as the substrate for FAO, so we subsequently determined whether Acox1-mediated FAO involved in ISCs proliferation was enhanced by CBD. As expected, the inhibitory rate of FAO in intestinal crypts damaged by IR could be improved by CBD, and the suppression of Fatp2 with lipofermata abolished the stimulatory effect of CBD on the FAO rate (Fig. 3h). In parallel, pharmacological disruption of Acox1 with 10,12-tricosadiynoic acid^38^ obviously counteracted ISCs proliferation triggered by CBD/exogenous FAs complement or *Slc27a2* overexpression upon IR injury (Fig. 3g,j and Supplementary Figs. 5g,h and 6a–c), Since FAs ultimately undergo FAO in the mitochondria to generate substrates for energy metabolism^9,10^, we determined the mitochondrial membrane potential in organoids to investigate whether CBD could enhance the mitochondrial activity. Our results showed that CBD could restore the mitochondrial membrane potential reduced by IR injury, while the lipofermata counteracted the effect of CBD (Fig. 3i). The above results indicated that FA transport mediated by FATP2 coupled with FAO initiated by Acox1 was crucial for CBD-favored ISCs proliferation post IR.

### PPARα serves as a master regulator of FAO triggered by CBD

**Fig. 4:**
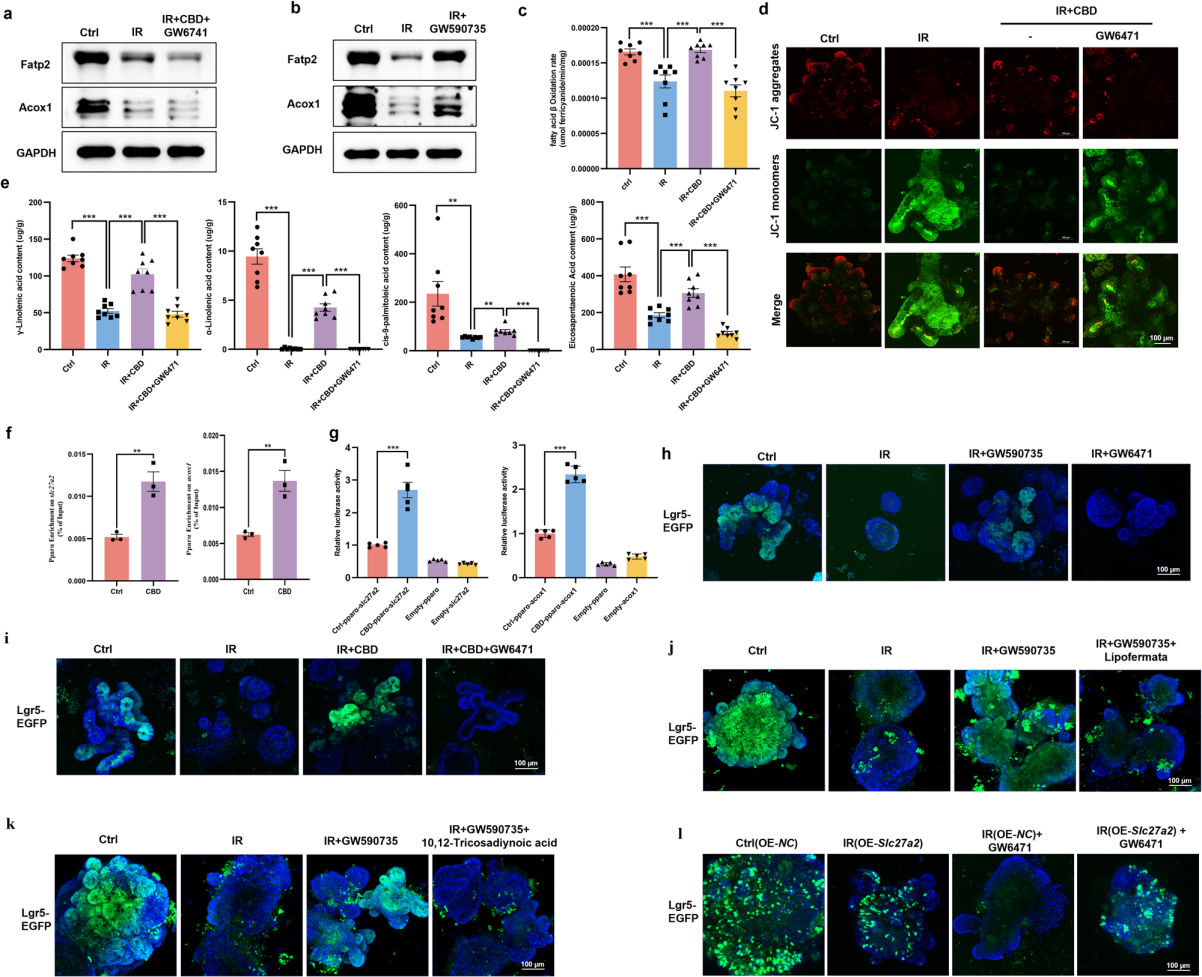
PPARα is a predominant regulator of the CBD-mediated FAO program during IR damage. **a**, **b** With intestinal organoids, the PPARα antagonist GW6471 (4 μM) was found to abolish the effect that CBD rescued Fatp2 and Acox1 protein levels after IR damage (**a**), while the PPARα agonist GW590735 (1 nM) restored Fatp2 and Acox1 protein levels decreased by IR (**b**). **c**–**e** GW6471 abolished the effect that CBD rescued the decreased rate of FAO in crypts (**c**), decreased levels of mitochondrial membrane potential in intestinal organoids (**d**) and exogenous FAs in crypts (**e**) caused by IR. **f**, **g** CBD stimulated the transcriptional activation of *Slc27a2* and *Acox1* by PPARα with ChIP (**f**) and dual-luciferase assays (**g**). **h**, **i** GW590735 triggered Lgr5^+^ ISCs proliferation (**h**) while GW6471 inhibited the radioprotective effect of CBD on ISCs (**i**). **j**, **k** The proliferation of Lgr5^+^ ISCs triggered by GW590735 was inhibited by lipofermata (**j**) or 10,12-tricosadiynoic acid (**k**). **l** GW6471 counteracted the effect of *Slc27a2* overexpression on ISCs proliferation during IR damage. The results are expressed as the mean ± s.d. **P < 0.01 and ***P < 0.001.

**Fig. 5:**
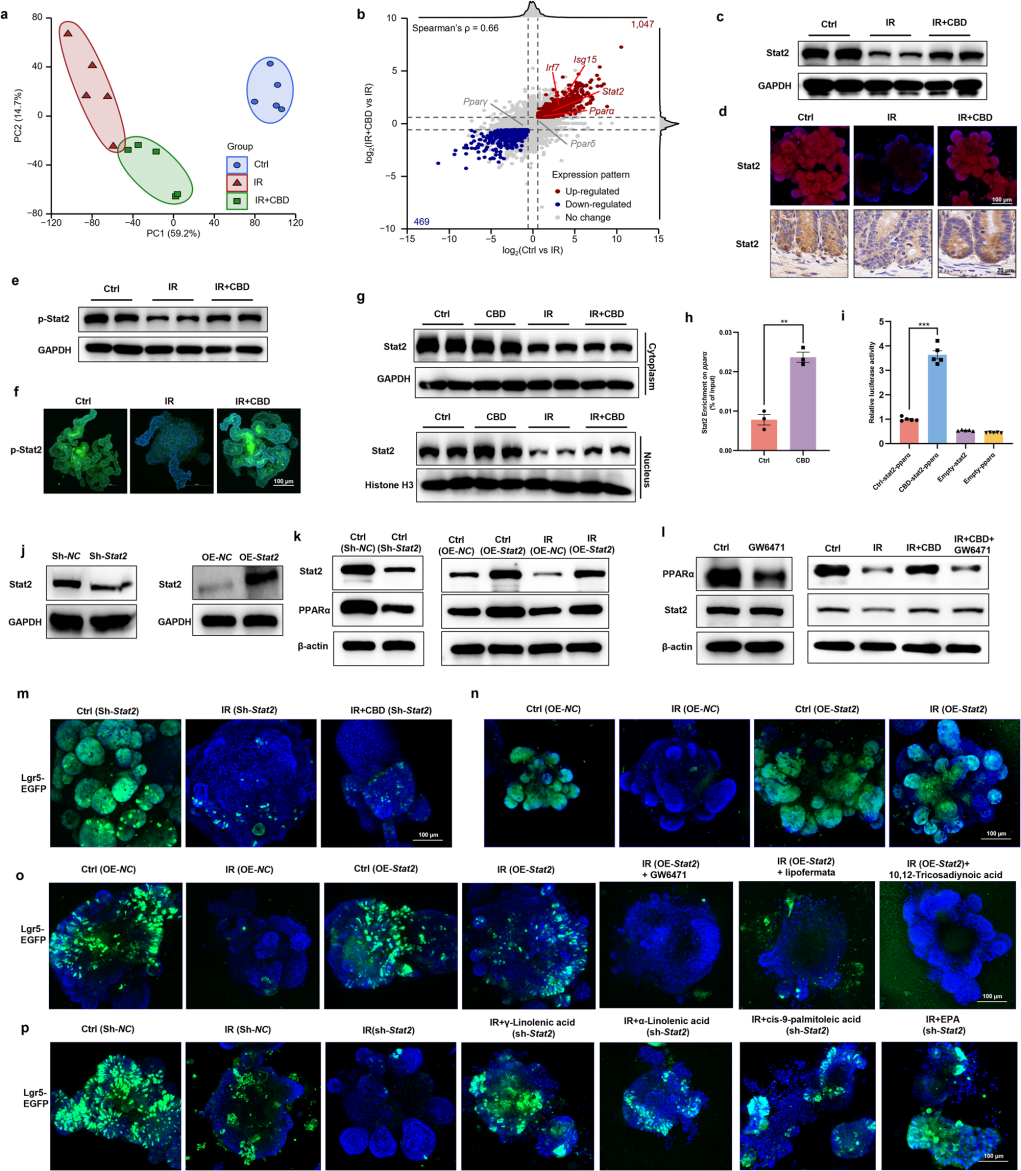
Stat2 serves as an upstream regulator to facilitate PPARα-mediated ISCs regeneration upon CBD treatment. **a** PCA based on the transcriptomes of the Ctrl, IR and IR + CBD groups. **b** A scatter plot showing the log_2_FC of genes in the Ctrl versus IR and IR + CBD versus IR comparisons. **c**, **d** The protein levels of Stat2 were detected with a western blot assay (**c**) and visualized by IF staining in intestinal organoids or IHC in small intestine (**d**). **e**, **f** The protein level of p-Stat2 was detected with a western blot assay (**e**) and visualized by IF staining in intestinal organoids (**f**). **g** The nuclear translocation of Stat2 was determined with a western blot assay. **h**, **i** The CBD-stimulated transcriptional activation of *Pparα* by Stat2 with ChIP (**h**) and dual-luciferase assays (**i**) was determined. **j**, **k** The knock down of *Stat2* (**j**) in intestinal organoids caused the downregulation of PPARα (**k**), while *Stat2* overexpression (**j**) upregulated PPARα protein expression with or without IR damage (**k**). **l** GW6471 did not influence Stat2 protein expression. **m**, **n** The knock down of *Stat2* abolished the protective effect of CBD on ISCs (**m**), while *Stat2* overexpression triggered Lgr5^+^ ISCs proliferation (**n**) upon IR exposure. **o** GW6471, lipofermata or 10,12-tricosadiynoic acid markedly counteracted the effect of *Stat2* overexpression in rescued ISCs proliferation disrupted by IR. **p** The ablation of *Stat2* did not influence the radioprotective effects of exogenous FAs on ISCs. The results are expressed as the mean ± s.d. **P < 0.01 and ***P < 0.001.

mRNA sequencing and subsequent western blot/immunohistochemistry assays showed that PPARα was stimulated by CBD upon IR injury (Fig. 5b and Supplementary Fig. 7a–c), and it seems that PPARα played a key role in regulating the genes mediating lipid metabolic remodeling involved in the radioprotective activity of CBD (Fig. 3c). Integrated bioinformatics analyses of transcriptome and lipidomics was therefore used to determine the correlation between the 14 DEGs and 28 DALs identified above (Figs. 2c and 3c). After applying a filtering criteria of Spearman’s *ρ* > 0.5 and *P* value <0.05, a total of 110 gene–lipid pairs consisting of 14 DEGs and 11 DALs were identified, in which all the 11 DALs were those upregulated lipids triggered by CBD upon IR injury (Supplementary Fig. 7d), indicating that PPARα was indispensable for FA metabolism modulated by CBD. As expected, pharmacologic disruption of PPARα with the antagonist GW6471 (ref. ^39^) diminished the ability of CBD to restore the protein levels of Fatp2 and Acox1, FA content, FAO rate and the mitochondrial membrane potential downregulated by IR challenge (Fig. 4a,c–e). ChIP and dual-luciferase assays showed that CBD evoked the transcription of *Slc27a2* and *Acox1* regulated by PPARα (Fig. 4f,g), which was consistent with the result that the PPARα agonist GW590735 (ref. ^40^) stimulated the upregulation of Fatp2 and Acox1 (Fig. 4b).

We next assessed the critical function of PPARα on CBD-driven ISC survival in response to IR challenge. Pretreatment of GW590735 mimicked the radioprotective activity of CBD, in which the aberrant budding and ISCs loss in intestinal organoids caused by IR were ameliorated with GW590735 (Fig. 4h and Supplementary Fig. 8a,b). Conversely, GW6471 treatment aggravated IR-induced injury on intestinal organoids, in parallel with the abrogation of the radioprotective effect of CBD (Fig. 4h,i and Supplementary Fig. 8a–d). To investigate whether the transactivation of *Slc27a2* and *Acox1* by PPARα mitigated the proliferation inhibition of ISCs evoked by IR, gain-of-function and loss-of function analyses were used. The results showed that pharmacologic disruption of Fatp2 or Acox1 apparently counteracted the ability of the PPARα agonist to trigger ISCs proliferation upon IR injury, while the overexpression of *Slc27a2* could alleviate the aggravation of ISCs loss caused by the PPARα antagonist (Fig. 4j–l). Further, although the radioprotective effect of CBD on ISCs was abrogated by the PPARα antagonist, supplementation with exogenous FAs could remarkably compensate for ISCs proliferation (Supplementary Fig. 8e). These data suggest that CBD facilitated the binding of PPARα to the promoters of *Slc27a2* and *Acox1*, thereby stimulating their transcription, and the upregulation of FATP2 and Acox1 would subsequently favor FAO and ISCs proliferation upon IR exposure.

### Stat2 upregulation triggered by CBD favors PPARα-mediated ISCs proliferation upon IR exposure

A high Spearman’s correlation coefficient (*ρ* = 0.66) was obtained from the differential gene expression analysis, and the signaling response to interferon-β (primary DEGs were *Stat2*, *isg15* and *irf7*) inhibited by IR was found to be rescued by CBD (Fig. 5a,b). Consistently, CBD was found to maintain the protein expression levels of Stat2 in the intestinal crypts or organoids (Fig. 5c,d). Given that *isg15* and *irf7* are target genes of Stat2, we hypothesized that CBD might maintain the transcriptional activity of Stat2 during IR damage, which would then stimulate the signaling mediated by PPARα. We first determined the stimulation of Stat2 and the results showed that the phosphorylated levels and nuclear translocation of Stat2 reduced by IR was apparently rescued by CBD (Fig. 5e–g). Further, the ChIP assay revealed that CBD could trigger the enrichment of Stat2 on the promoter of *Pparα*, which was further verified using luciferase reporter assays (Fig. 5h,i). Furthermore, protein levels of PPARα were markedly decreased in intestinal organoids deficient in Stat2, while *Stat2* overexpression rescued the levels suppressed by IR (Fig. 5j,k). Nevertheless, the PPARα antagonist showed no impact on Stat2 expression with or without CBD treatment upon IR injury (Fig. 5l). These results imply that the activation of PPARα signaling in ISCs stimulated by CBD during IR damage was mediated by Stat2.

Considering the importance of Stat2 on PPARα signaling modulated by CBD, we next investigated the essential role of Stat2 in regulating ISCs proliferation. Stat2 deficiency dramatically counteracted the proliferation of ISCs stimulated by CBD during IR injury, while the ectopic expression of Stat2 in intestinal organoids markedly sustained ISCs homeostasis post IR, indicating the indispensable role of Stat2 on the radioprotective activity of CBD (Fig. 5m,n). PPARα signaling was then disrupted with a selective antagonist and ISCs proliferation in organoids overexpressing Stat2 post IR was found to be compromised (Fig. 5o), which was coincident with the transcriptional activation of *Pparα* by Stat2. In parallel, the Fatp2 or Acox1 inhibitors obviously abolished the proliferation of ISCs stimulated by the ectopic expression of Stat2, while the deficiency of Stat2 did not influence the protective effects of exogenous FAs against IR toxicity on ISCs (Fig. 5o,p). Together, our data demonstrate that CBD facilitated the transcriptional upregulation of PPARα by Stat2, which then stimulated the protein expression of Fatp2 and Acox1, thus favoring ISCs proliferation post IR.

### THOC3 was identified as a unique target of CBD

**Fig. 6:**
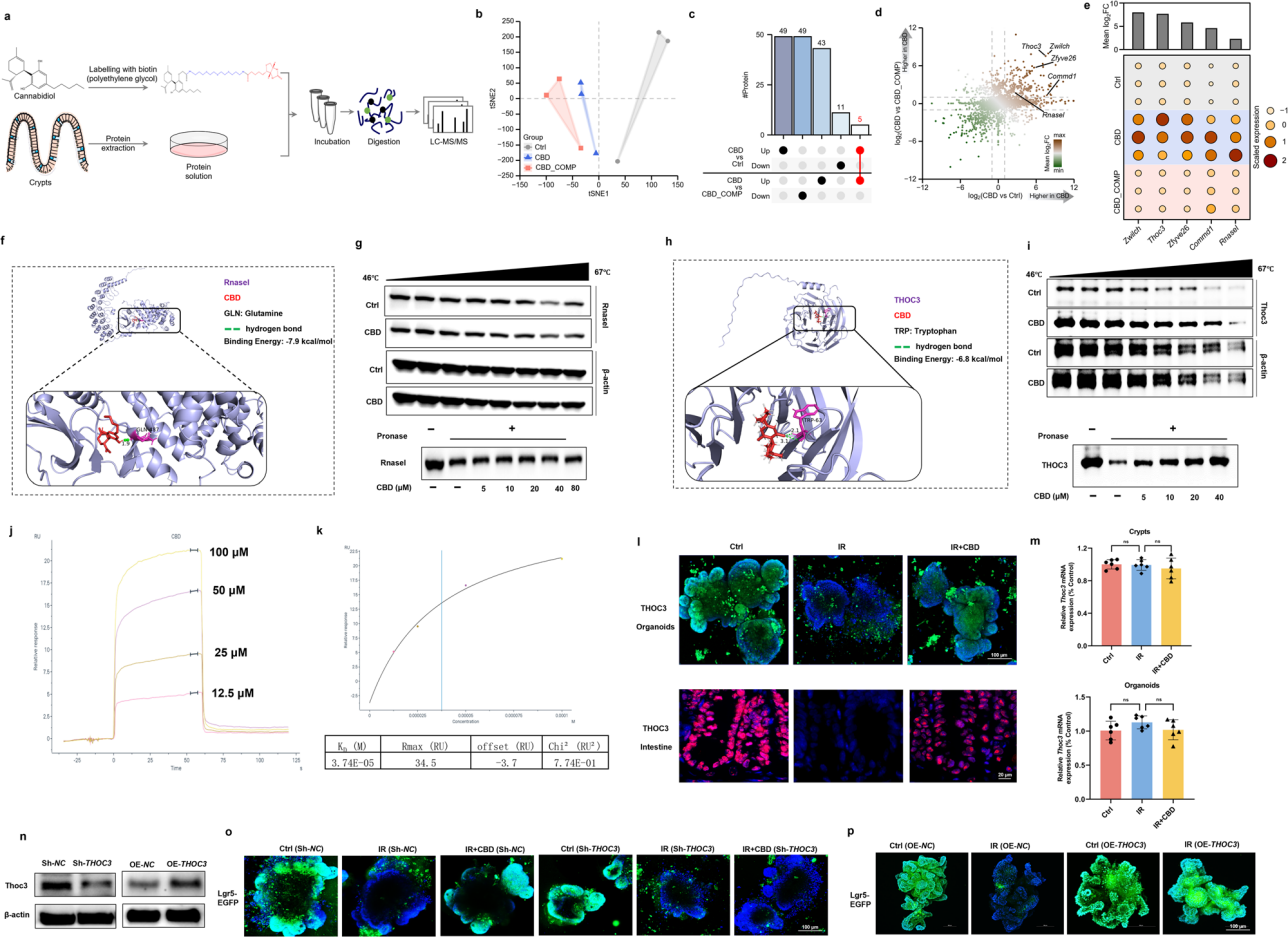
THOC3 is the direct target of CBD in favoring ISC proliferation during IR damage. **a** A scheme of CBD molecular probe (Biotin-CBD) synthesis and pull down of the CBD-binding conjugate by proteomics. **b** A scatter plot showing the *t*-SNE results of the protein expression levels in Ctrl, CBD and CBD_COMP groups. **c** An upset plot showing the number of DEPs between the CBD versus Ctrl and CBD versus CBD_COMP comparisons. The DEPs that were common to both comparisons are highlighted in red. **d** A scatter plot displaying the expression changes of the identified proteins in the Ctrl, CBD and CBD_COMP groups. **e** A dot plot displaying the expression pattern of five upregulated proteins that were commonly identified in both CBD versus Ctrl and CBD versus CBD_COMP comparisons. **f**–**i** Molecular docking analysis of the binding between CBD and RNasel (**f**), CETSA and DARTS assays verifying the interaction of CBD with RNasel (**g**); molecular docking analysis of the binding between CBD and THOC3 (**h**), CETSA and DARTS assays verifying the interaction of CBD with THOC3 (**i**). **j** SPR analysis of the binding affinity of CBD to THOC3 protein. **k** The apparent equilibrium dissociation constants (*K*_d_) were calculated. **l** The protein levels of THOC3 in intestinal organoids and small intestine were detected by IF. **m** *THOC3* mRNA expression in intestinal crypts or organoids was detected by qRT–PCR. **n**–**p** Lentiviral infection was applied to generate small intestinal organoids with stable *THOC3* knockdown (Sh‑*THOC3*) or *THOC3* overexpression (OE‑*THOC3*), respectively (**n**), Lgr5^+^ ISCs proliferation triggered by CBD was diminished when THOC3 was knocked down (**o**), while *THOC3* overexpression favored ISCs proliferation upon IR damage (**p**). The results are expressed as the mean ± s.d. ns, no significance.

To identify the direct target of CBD, a CBD molecular probe (Biotin-CBD) was synthesized by introducing a biotin side chain linked with polyethylene glycol (PEG) (Fig. 6a). A PEG chain was used to enhance the spatial distance between biotin and CBD, and biotin could bind to streptavidin agarose, which was then incubated with proteins extracted from crypts to pull down the cellular target of CBD. To eliminate the nonspecific binding proteins, ten times the concentration of CBD was used for the competitive combination with Biotin-CBD. Proteins in streptavidin agaroses obtained from the Biotin-captured group (Ctrl), Biotin-CBD-captured group (CBD) and the competitive binding group (CBD_COMP) were subjected to label-free proteomics analysis. *T*-distributed stochastic neighbor embedding (*t*SNE) analysis showed that there were significant differences among the three groups (Fig. 6b): 49 proteins were upregulated and 11 proteins downregulated in the Biotin-CBD-captured group as compared with the Biotin-captured group (CBD versus Ctrl), while 43 proteins were upregulated and 49 proteins downregulated in the Biotin-CBD-captured group as compared with the competitive binding group (CBD versus CBD_COMP) (Fig. 6c). Five proteins including THOC3, Commd1, Rnasel, Zfyve26 and Zwilch were ultimately screened as the potential pharmacological targets of CBD (Fig. 6d).

We analyzed the expression levels of the aforementioned proteins and found that THOC3 and Zwilch were the top two in terms of abundance (Fig. 6e). Further analysis of their intracellular localization revealed that Zwilch is primarily distributed at chromosomal centromeres, while THOC3 is localized in the nucleus. THOC3 is critical for efficient mRNA export, thereby coupling gene transcription with RNA processing^41^, and thus THOC3 is more suitable as a protein for regulating downstream signaling pathways. Next, we conducted virtual docking experiments and the results showed that the binding affinities ranked as follows: Rnasel (−7.9 kcal/mol) > THOC3 (−6.8 kcal/mol) > Zwilch (−6.5 kcal/mol) > Zfyve26 (−6.1 kcal/mol) > Commd1 (−5.5 kcal/mol) (Fig. 6f,h and Supplementary Fig. 9). To investigate whether Rnasel could bind to CBD, we performed CETSA and DARTS assays, which revealed that CBD does not bind to Rnasel (Fig. 6g).

Indeed, the CETSA assay revealed that CBD treatment could protect THOC3 from temperature-dependent degradation, in parallel with the resistance to proteolysis with the DARTS assay (Fig. 6i). SPR results showed that CBD directly interacted with THOC3 in a positive dose-dependent manner and the determined equilibrium dissociation constant (*K*_d_) for CBD binding to THOC3 was 37.4 μM (Fig. 6j,k). The *K*_d_ value reflects the strength of binding between a drug and a protein at equilibrium and our results indicate that only 37.4 μM of CBD is needed to occupy a large fraction of the THOC3 protein. Further, CBD treatment enhanced the stability of THOC3 during IR damage, in which the decreased expression of THOC3 in intestinal organoids or epithelium caused by IR were alleviated upon CBD treatment (Fig. 6l). As CBD had only a minor effect on the transcription of *THOC3* (Fig. 6m), CBD might sustain the stability of THOC3 protein, thereby conferring radioresistance to ISCs. Then, *THOC3* was ablated or overexpressed in intestinal organoids (Fig. 6n) and ISC proliferation stimulated by CBD post IR damage was ultimately compromised in *THOC3*-depleted organoids (Fig. 6o), while *THOC3* overexpression profoundly protected ISCs against IR damage (Fig. 6p).

### The direct targeting of CBD to THOC3 supports Stat2 mRNA nuclear export

**Fig. 7:**
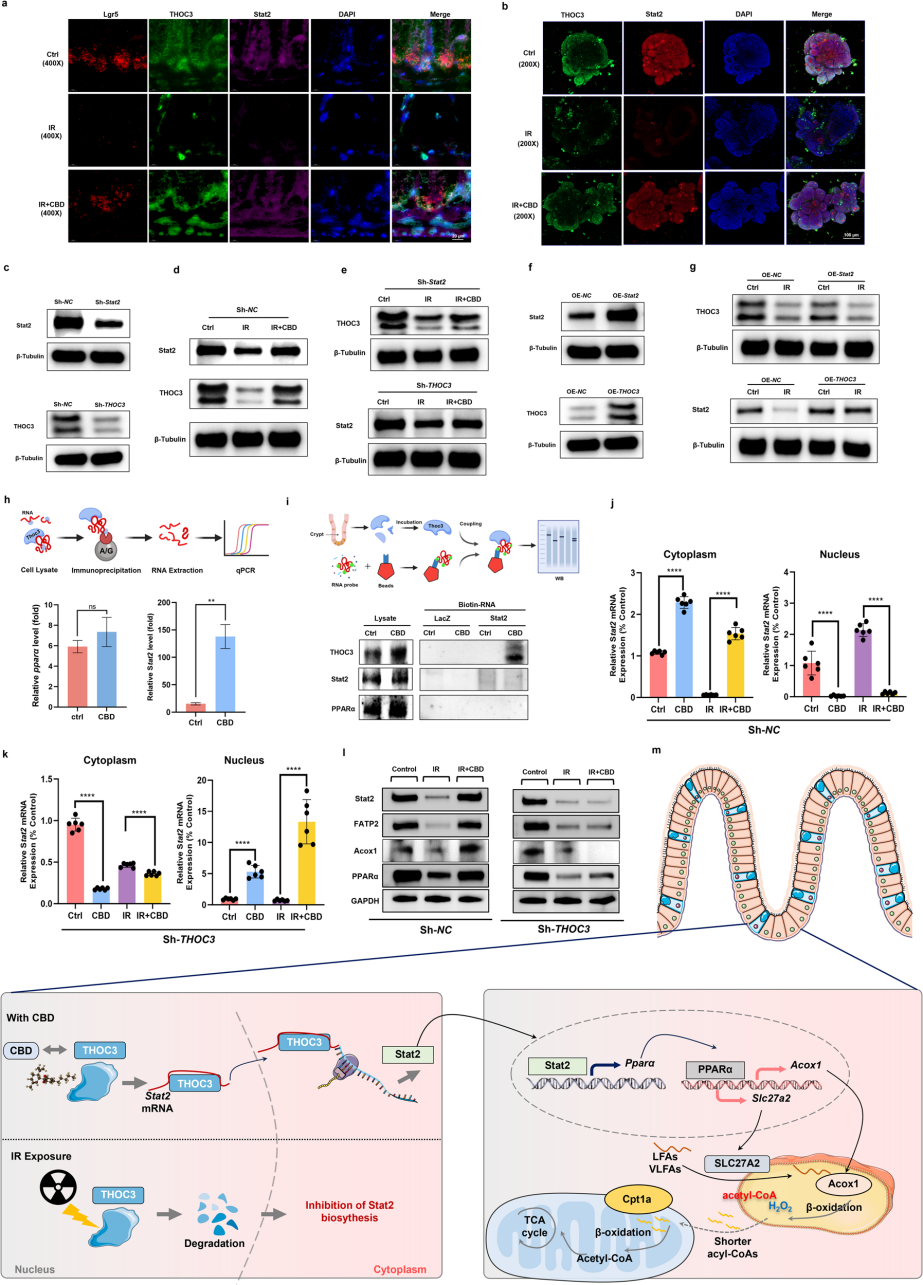
Radiation stability of THOC3 with CBD treatment relieved the retardation of Stat2 mRNA in the nucleus. **a** The colocalization of THOC3 and Stat2 mRNA in Lgr5^+^ ISCs with the RNAscope assay. **b** The changes in the level of THOC3 with or without CBD treatment upon IR exposure were consistent with those of Stat2 in intestinal organoids by IF. **c**–**e** *Stat2* or *THOC3* in intestinal organoids were knocked down (**c**) and the protein levels of THOC3 or Stat2 triggered by CBD upon IR exposure were determined by western blot (**d** and **e**). **f**, **g** *Stat2* or *THOC3* in intestinal organoids were overexpressed (**f**) and the protein levels of THOC3 or Stat2 were determined by western blot (**g**). **h**, **i** The enrichment of PPARα or Stat2 mRNA in THOC3 immunoprecipitates upon CBD treatment by qRT–PCR (**h**) and the direct binding between THOC3 and Stat2 mRNA was confirmed with an RNA pulldown assay (**i**). **j**, **k** Detection of *Stat2* mRNA expression in the cytoplasm and nucleus of Sh-*NC* (**j**) or Sh-*THOC3* (**k**) cells by RT-qPCR. **l** The protein level of Stat2, PPARα, Fatp2 and Acox1 rescued by CBD upon IR damage was mediated by THOC3. **m** A schematic overview of the intrinsic mechanism mediating the proliferation of ISCs triggered by CBD upon IR exposure. The results are expressed as the mean ± s.d. **P < 0.01 and ****P < 0.0001. ns, no significant.

As the change in the levels of THOC3 with or without CBD treatment upon IR exposure was consistent with that of Stat2, and THOC3 and Stat2 were both found to be colocalized in Lgr5^+^ ISCs with the RNAscope assay (Fig. 7a,b), we then performed experiments to determine whether the radiation stability of THOC3 contributed to Stat2-mediated ISCs proliferation post IR. Using lentiviruses for the knockdown of *THOC3* or *Stat2* (Fig. 7c), we found that *THOC3* ablation abolished the effect that CBD rescued Stat2 protein expression after IR damage, whereas knockdown of *Stat2* had no apparent effect on THOC3 protein levels (Fig. 7d,e). Similarly, *THOC3* overexpression alleviated the degradation of Stat2 protein following IR damage, while the overexpression of *Stat2* had no apparent effect on THOC3 protein expression (Fig. 7f,g). The above experiments demonstrated that THOC3 serves as an upstream regulator to favor the expression of Stat2 upon CBD treatment. Given that THOC3 was demonstrated to favor mRNA nuclear export, we first determined the interaction of THOC3 with Stat2 mRNA and found that CBD treatment could trigger the binding of THOC3 to Stat2 mRNA, but not PPARα mRNA, using the RNA immunoprecipitation assay (Fig. 7h). Then a biotin-labeled Stat2 mRNA probe was constructed and an RNA pulldown assay was used. The results confirmed that CBD indeed obviously promoted the direct binding between Stat2 mRNA and the THOC3 protein (Fig. 7i).

Subsequent experiments showed that, after IR injury, the level of Stat2 mRNA in the nucleus elevated significantly but it decreased in the cytoplasm, and the reverse tendency was observed with CBD treatment (Fig. 7j). However, *THOC3* ablation impeded the nuclear export of Stat2 mRNA facilitated by CBD during IR damage, which was consistent with the abolition of CBD in saving PPARα, Fatp2 and Acox1 protein expression (Fig. 7l). Our results demonstrate that CBD targeting of THOC3 relieves the blockade of Stat2 mRNA nuclear export during IR injury, thereby stimulating PPARα-mediated expression of Fatp2 and Acox1 (Fig. 7m).

## Discussion

There is currently no effective countermeasure for IR-induced GIS; as ISCs are the primary source for intestinal regeneration, the timely proliferation of ISCs restoring the ability of intestinal crypts to repair intestinal epithelium is one potential strategy. In the current study, the radioprotective activity and the intrinsic mechanism of CBD against intestinal damage was investigated. CBD treatment was found to improve the survival rate and body weight loss of mice, maintain the multiplication capacity of crypt cells and trigger the regeneration of Lgr5^+^ ISCs post IR exposure (Fig. 1), suggesting that the stimulation of ISCs proliferation was involved in the radioprotective activity of CBD against GIS. Intriguingly, our results showed that the radioprotective effect of CBD on ISCs was superior to amifostine (WR-2721), which is the radiomitigator approved by the FDA for clinical use. Currently, apart from six growth factors or cytokines, only amifostine has been approved by the US FDA as a protective drug for IR-induced injury^42^ but its dose-dependent toxic side effects and the strict administration schedule have imposed serious restrictions on its application^43^. The potent radioprotective effect and very low toxicity of CBD point to its promise as a radioprotective agent for further development.

Lipid metabolism is crucial for modulating stem cell state and differentiation, but lipid mobilization and utilization in stem cell-driven regeneration upon IR damage remain unclear^9^. Lipid metabolism deregulation was observed in crypt cells exposed to IR damage with lipidomics sequencing, and CBD treatment could relieve the disturbance on lipid metabolism. Among the 14 downregulated lipids that were reversed by CBD, 10 of them belong to PEs, so we analyzed the lipid metabolic program of these PEs with LMSD and found that these PEs can be further metabolized into long-chain FAs (LCFAs; γ-linolenic acid, α-linolenic acid and *cis*-9-palmitoleic acid) and a very-long-chain FA (VLCFA; EPA) (Fig. 2c). The exogenous supply of these FAs substantially stimulated the proliferation of ISCs after IR injury (Fig. 2f,g). Correspondingly, pathway enrichment analysis with GO and KEGG reveal that the inhibition of FA metabolic process, lipid catabolic process and FA degradation could be relieved with CBD treatment upon IR exposure (Fig. 3a,b). The ablation of lipolysis or FA synthesis pathway was reported to cause ISCs impairment^7,18^, and our results indicate that CBD could trigger the proliferation of ISCs during IR exposure by modulating FA metabolism.

To unveil the molecular networks underlying ISCs-driven intestinal regeneration with CBD treatment post IR damage, mRNA transcriptomics was used. The results show that CBD treatment upon IR exposure markedly upregulated genes involved in fatty acid transport (*Slc27a2*, *CD36* and *Slc27a4*) and subsequent β-oxidation (*Acaa1a*, *Acox1*, *Acox3*, *Ehhadh* and *Scp2*) (Fig. 3c). Fatp2 encoded by *Slc27a2* was localized in the peroxisome, endoplasmic reticulum and cell membrane, which can drive FA uptake by supporting their transportation across plasma membrane or serving as an acyl-CoA ligase to catalyze LCFA/VLCFA into fatty acyl-CoA^44,45^. A previous study demonstrated that the activation of *Slc27a2* by HNF4 facilitated the self-renewal of ISCs^14^ and our results showed that the overexpression of *Slc27a2* prevented the depletion of ISCs, while the inhibition of Fatp2 abolished the protective effects of CBD on ISCs upon IR damage (Fig. 3f, j).Notably, all CBD-activated genes involved in FAO were associated with peroxisomal β-oxidation. Acox1, the first rate-limiting enzyme in this pathway, catalyzes the desaturation of fatty acyl-CoAs derived from LCFA/VLCFA into *trans*-2-enoyl-CoAs, generating hydrogen peroxide (H₂O₂) in the process^10,35^. The resulting chain-shortened acyl-CoAs can subsequently be converted into carnitine esters (acylcarnitines) for mitochondrial FAO^46^. As our results show, CBD treatment rescued the FAO rate in crypts post IR injury, and the inhibition of Acox1 abolished ISCs proliferation triggered by CBD/exogenous FA treatment or *Slc27a2* overexpression (Fig. 3g–j and Supplementary Fig. 6). As inhibition of Fatp2 counteracted CBD-induced FAO stimulation upon IR exposure (Fig. 3h), activation of Slc27a2—which promotes fatty acid transport coupled to Acox1-mediated peroxisomal FAO—may contribute to the radioprotective effect of CBD on ISCs.

PPARα, a nuclear receptor subfamily 1 group C member 1 (NR1C1) crucial for peroxisomal FAO, FA transport and ketogenesis^47^, was identified to be a master regulator of CBD-mediated lipid metabolism through the integrative analyses of transcriptome and lipidomics. Previous research has demonstrated that PPARα activated a robust Cpt1a-mediated FAO program for ISCs expansion^48^, while the suppression of PPARα impeded FAO or increased the Notum level, impairing Lgr5^+^ ISC renewal^15,49^. In our study, the activation of PPARα with CBD treatment was demonstrated to transactivate *Slc27a2* and *Acox1*, which subsequently restored FAO to improve Lgr5^+^ ISC proliferation during IR damage (Fig. 4).

Further transcriptomic analysis revealed that the interferon-β response pathway was dramatically altered by CBD treatment after IR damage, accompanied by concomitant changes in Stat2 levels within the JAK–STAT signaling pathway. JAK–Stat2 is vital for the cellular response to type I interferons, wherein the phosphorylation of Stat2 and Stat1 complex with IRF9 to form the heterodimer ISGF3 that accumulates in the nucleus to stimulate the transcription of interferon-stimulated genes (ISGs)^50^. Stat2 was found to favor lipid synthesis by upregulating acetyl-CoA carboxylase 1 (ref. ^51^), while the ablation of Stat2 resulted in the downregulation of the gene encoding fatty acid-binding protein 4 (ref. ^52^), indicating a vital role of Stat2 in lipid metabolism. We hypothesized that Stat2 might be involved in PPARα-mediated ISCs expansion triggered by CBD treatment. As expected, Stat2 was indispensable for ISCs proliferation and the radioprotective effect of CBD, wherein CBD first supported the transactivation of *Pparα* by Stat2, which then coupled the subsequent activation of *Slc27a2* and *Acox1* by PPARα to trigger the proliferation of ISCs during IR damage (Fig. 5).

To identify the direct molecular targets for CBD, protein extracts of intestinal crypts were incubated with biotinylated CBD or biotin, then streptavidin agarose beads was used to pull-down the conjugates for MS analysis, wherein THOC3 was found to be a binding partner of CBD (Fig. 6). THOC3 is a component of the THO subcomplex of the TREX complex mediating mRNA transcription, processing and nuclear export^53^. THO proteins have been found to modulate the proliferation and differentiation status of stem cells. THOC2 and THOC5 sustain the export and expression of pluripotency gene transcripts, thereby governing embryonic stem cell self-renewal and differentiation^53^, while THOC1 or THOC5 depletion led to the inhibition of ISC proliferation and the impairment of the gut epithelial barrier^54,55^. THOC3 has been reported to facilitate the transportation of mature mRNA from the nucleus to cytoplasm and is critical for the early stages of differentiation^41,56^; however, an essential role in modulating ISCs proliferation or self-renewal has not been reported. In the current study, CBD treatment was found to apparently trigger the binding of THOC3 with Stat2 mRNA to favor its nuclear export (Fig. 7h,i). The retardation of Stat2 mRNA in the nucleus relieved by CBD would ultimately sustain the transcriptional activity of PPARα, thereby triggering ISCs regeneration mediated by Fatp2 and Acox1 upon IR damage (Fig. 7j–l). Since THOC3 has been reported to mediate nuclear export of PFKFB4 mRNA in lung carcinoma cells, thereby facilitating proliferation^41^, CBD may target THOC3 to regulate additional mRNAs involved in signaling pathways that modulate ISCs proliferation; however, this requires further investigation.

In summary, the binding of CBD to THOC3 can maintain its radiation stability, which then supports the nuclear export of Stat2 mRNA for the subsequent transactivation of *Pparα.* The upregulated expression of PPARα will ultimately stimulate the FAO program mediated by Slc27a2 and Acox1, thereby facilitating ISCs proliferation during IR exposure (Fig. 7m).

## Supplementary information

Supplementary Information

## Data Availability

The data that support the findings of this study are available in the supplementary material of this article. The datasets generated during and/or analyzed during the current study are available from the corresponding author on reasonable request.

## Data availability

The data that support the findings of this study are available in the Supplementary Information of this Article. The datasets generated during and/or analyzed during the current study are available from the corresponding author on reasonable request. The RNA sequencing, lipidome, and label-free proteome data in this study are available at the China National Center for Bioinformation/National Genomics Data Center (CNCB/NGDC) under BioProject PRJCA058138, with dataset accessions CRA038715 (RNA-sequencing), OMIX015046 (lipidome), and OMIX015047 (proteome).
