# Supplementary material for: Cannabidiol triggers fatty acids β-oxidation mediated by Stat2 to facilitate intestinal stem cells regeneration post radiation
Source: Exp Mol Med. 2026 May 12;58(5):1642–56. doi: 10.1038/s12276-026-01711-5 (PMC13234352; doi:10.1038/s12276-026-01711-5)
Supplement: Supplementary file 1 — Supplementary Information [file 12276_2026_1711_MOESM1_ESM.pdf]

Supplementary Figure Legends

Supplementary Fig. 1 Cell apoptosis and DNA damage in situ were detected with Tunnel and γH2AX staining by IF (**a, b**). Panth cells labeled by lysozyme and ISCs labeled by SOX9 (**c-e**), $MUC2^+$ goblet cells (**f**) and $ChgA^+$ endocrine cells (**g**) were detected by IF. The schematic overview of experiment in which the mice were treated with agents post IR (**h**), and the change on intestinal structure (**i, j**) were determined with H&E staining. * $p<0.05$, ** $p<0.01$, *** $p<0.001$. ns, no significant.

Supplementary Fig. 2 Inflammatory response induced by IR damage was significantly inhibited by CBD. * $p<0.05$, ** $p<0.01$, *** $p<0.001$.

Supplementary Fig. 3 Dysregulation of lipid metabolism induced by IR was rescued by CBD.

Supplementary Fig. 4 Statistical analysis of EGFP fluorescence intensity, surface area and buds' numbers in intestinal organoids treated with exogenous FAs upon IR damage (**a**). Expression levels of mRNA mediating FAs transport and β-oxidation by qPCR (**b**). * $p<0.05$, ** $p<0.01$, *** $p<0.001$, **** $p<0.001$. $^{\#\#\#}$ $p<0.001$, $^{\#\#\#\#}$ $p<0.0001$ as compared to Ctrl group.

Supplementary Fig. 5 Fatp2 and Acox1 proteins expression in intestine or

organoids visualized with IF assay (**a-c**). Overexpression of *Slc27a2* in organoids upregulated Fatp2 protein expression (**d**). Radioprotective effect of CBD on ISCs was abolished by lipofermata (**e, f**) or 10,12-Tricosadiynoic acid (**h**). 10,12-Tricosadiynoic acid significantly counteracted the effect of *Slc27a2*-overexpression in rescued ISCs proliferation disrupted by IR (**g**).

Supplementary Fig. 6 Acox1 antagonist 10,12-Tricosadiynoic acid abolished the radioprotective effect of exogenous FAs on intestinal organoids and ISCs (**a-c**).

Supplementary Fig. 7 Changed levels of PPARα protein evoked by CBD upon IR exposure were determined by Western Blot (**a**), IHC (**b**) and IF (**c**) assay. The network map illustrated the expression correlation between DEGs and DALs. Green circles represent genes, and yellow squares represent lipids. The thickness of the connecting lines indicates the strength of the Spearman's correlation coefficient (ρ) between the two entities. Only the correlation relationships with Spearman's $\rho > 0.5$ and p-value < 0.05 were shown (**d**).

Supplementary Fig. 8 Effects of PPARα agonist GW590735 and antagonist GW6471 on intestinal organoids forming (**a**) and $Olfm4^+$ ISCs proliferation (**b**) upon IR damage. GW6471 significantly diminished the radio-protective effect of CBD on intestinal organoids (**c, d**), but did not influence the effect of

exogenous FAs (**e**).

Supplementary Fig. 9 Molecular docking assay were adopted to analyze the binding of CBD to Zwlich, Zfyve26 and COMMD1.

Supplementary Figures

Supplementary Fig. 1.

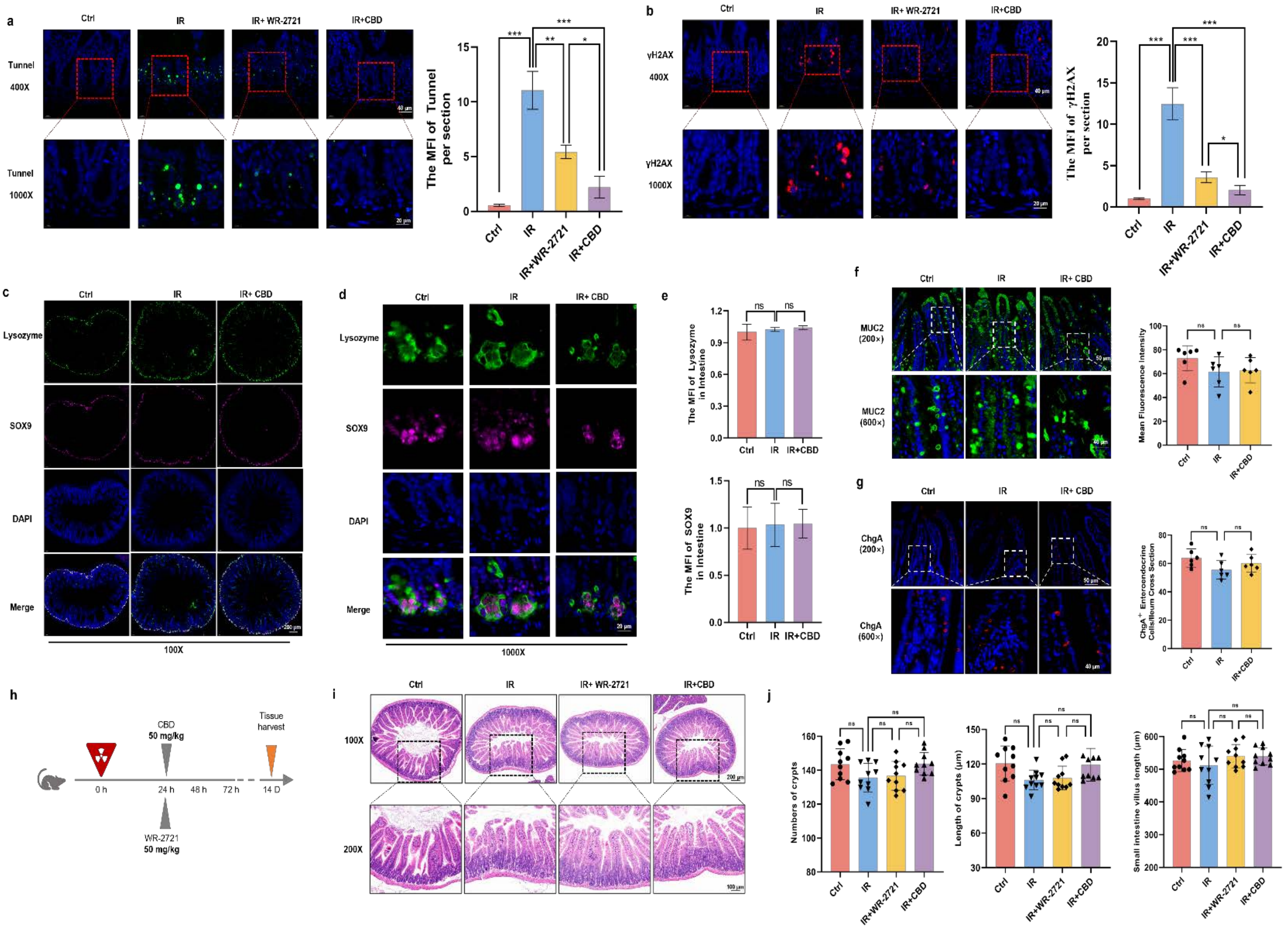

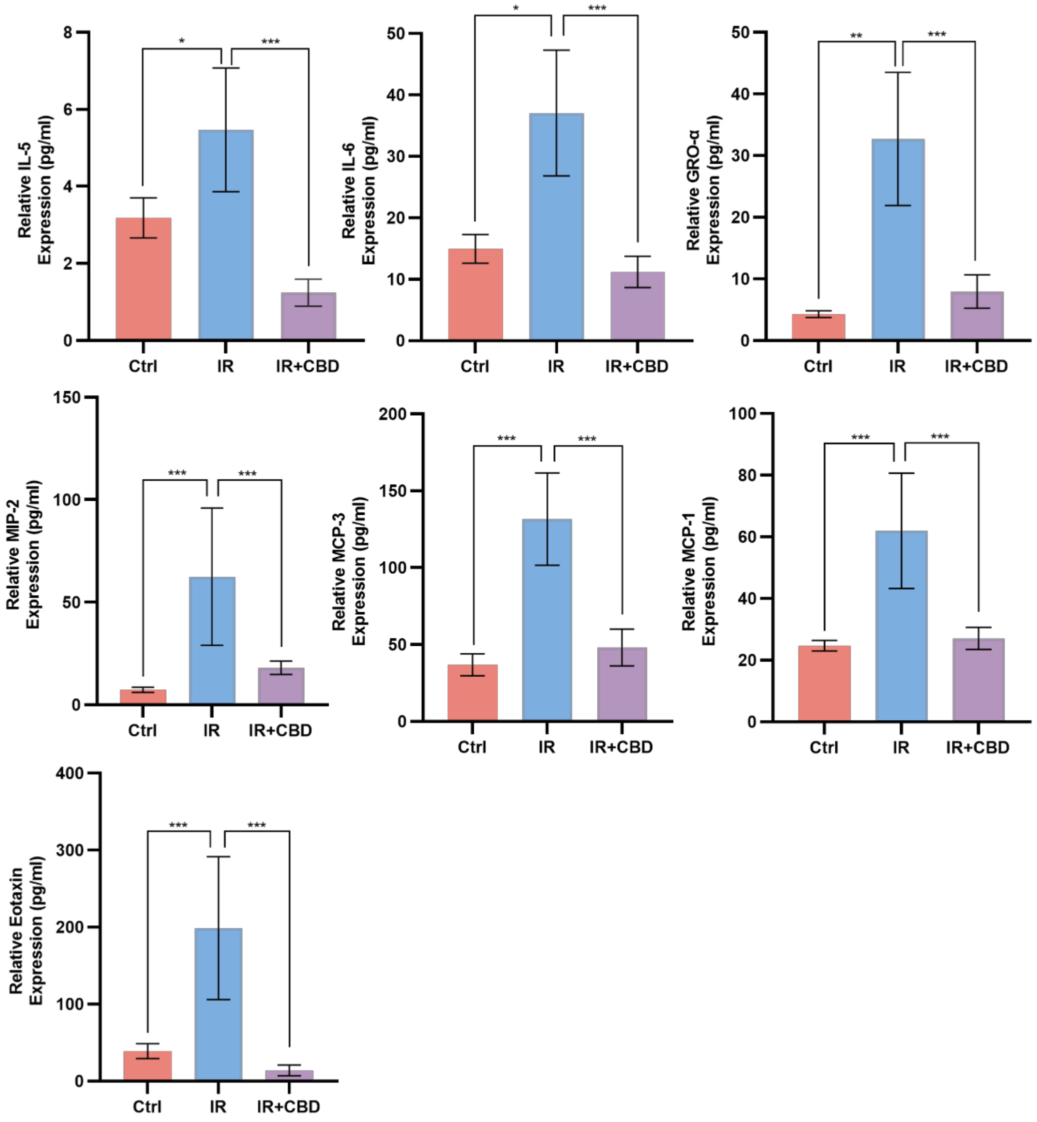

Supplementary Fig. 2.

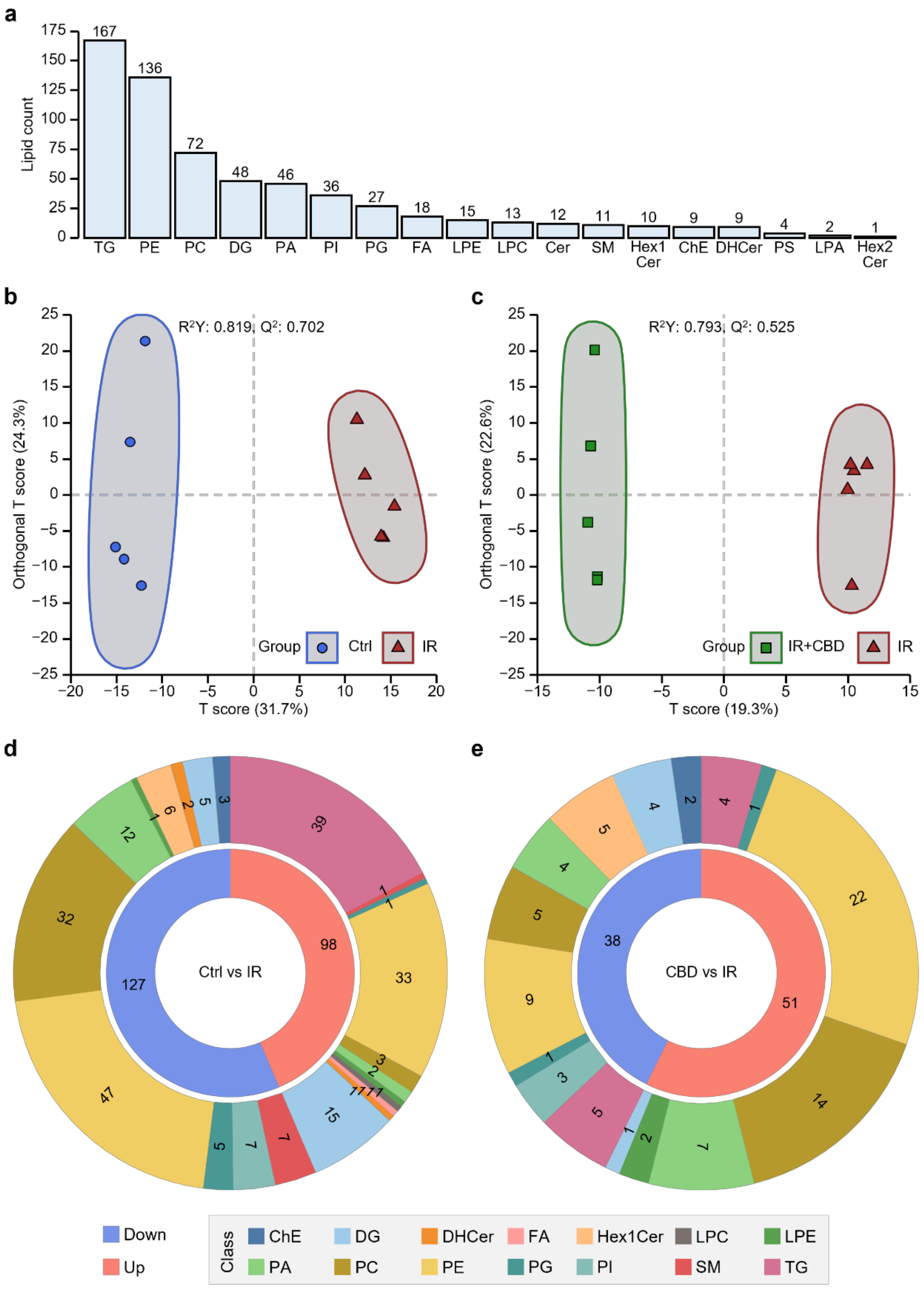

Supplementary Fig. 3.

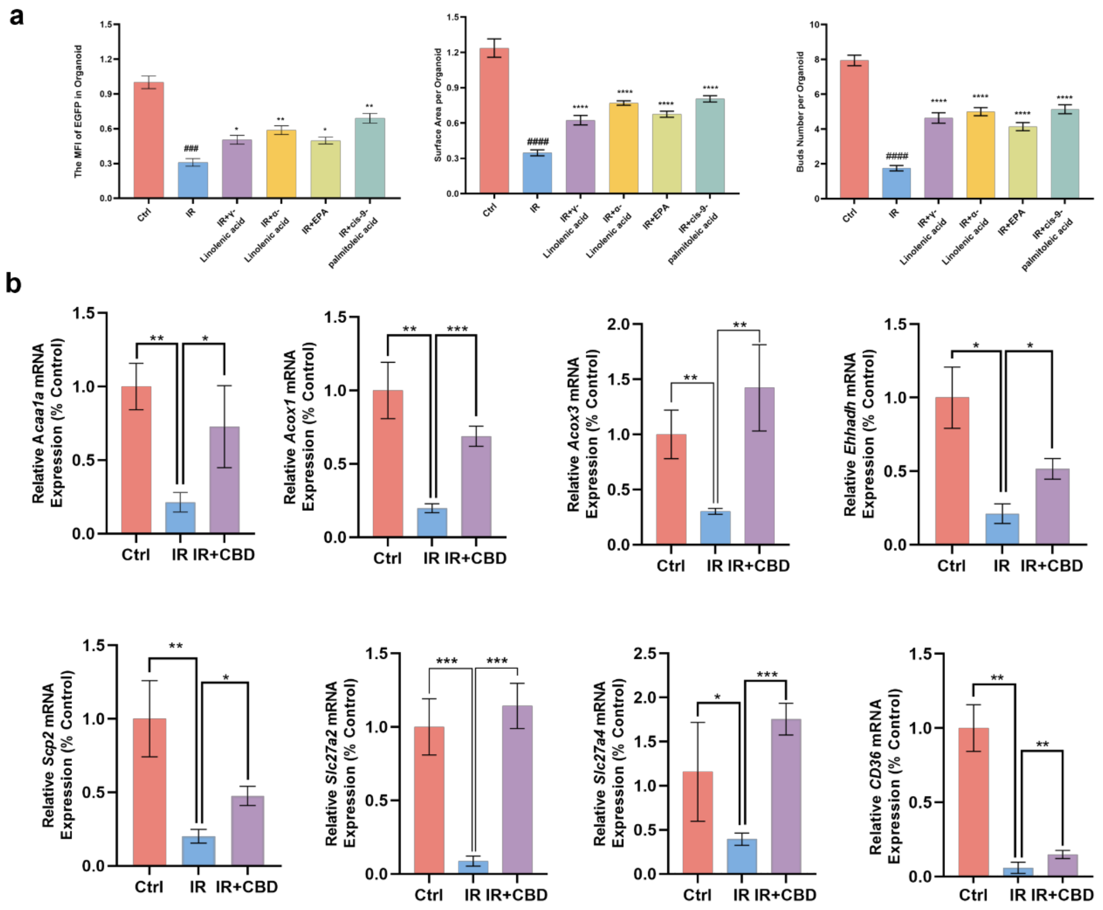

Supplementary Fig. 4.

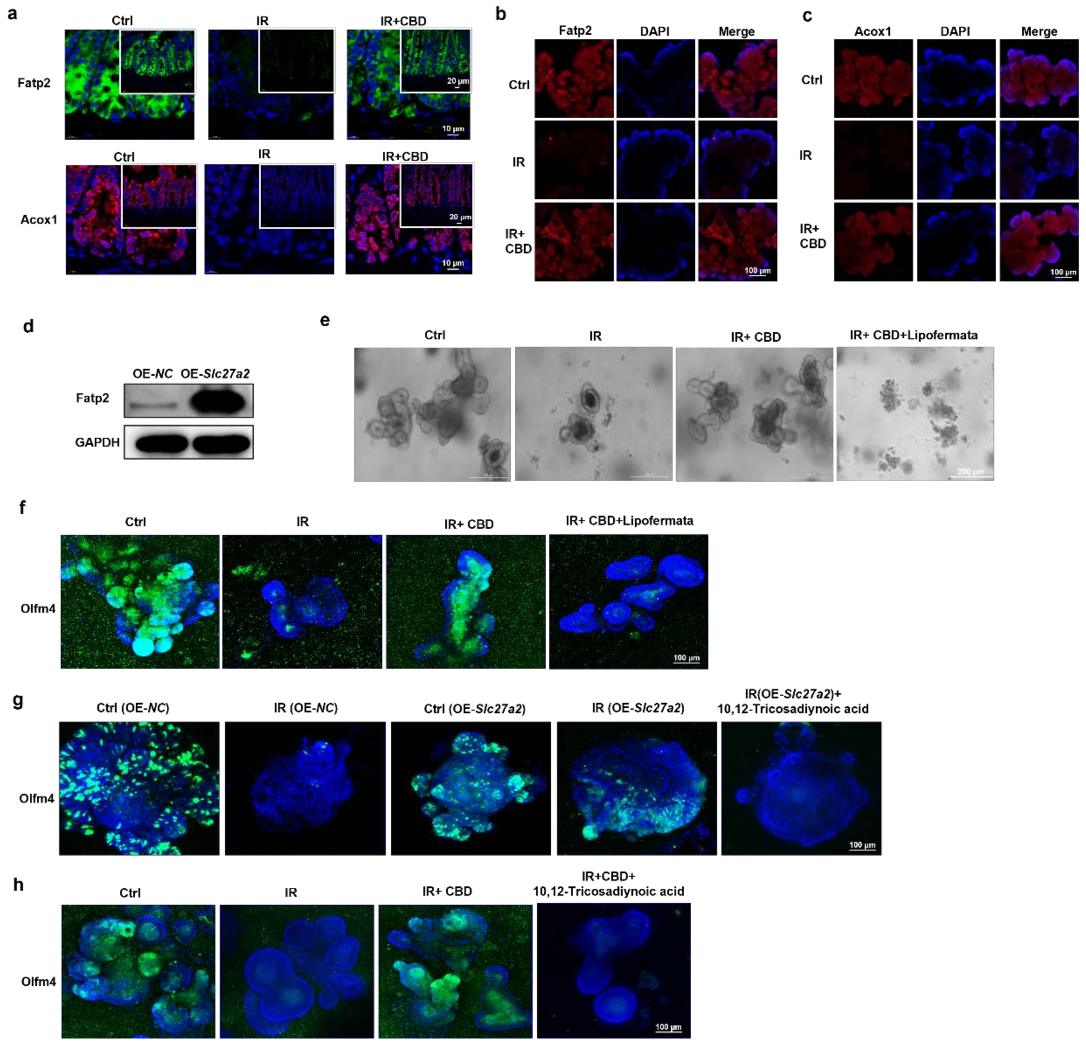

Supplementary Fig. 5.

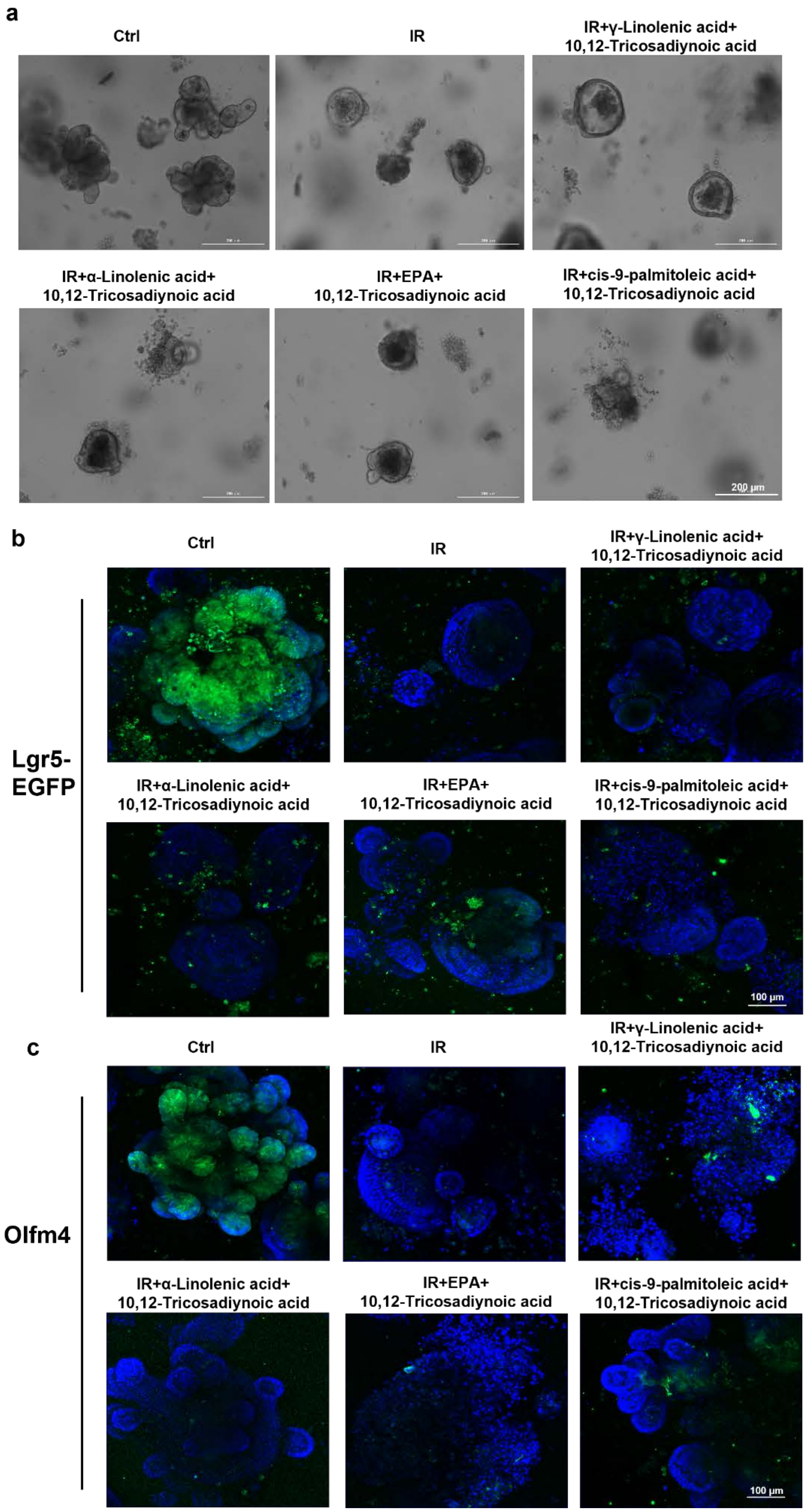

Supplementary Fig. 6.

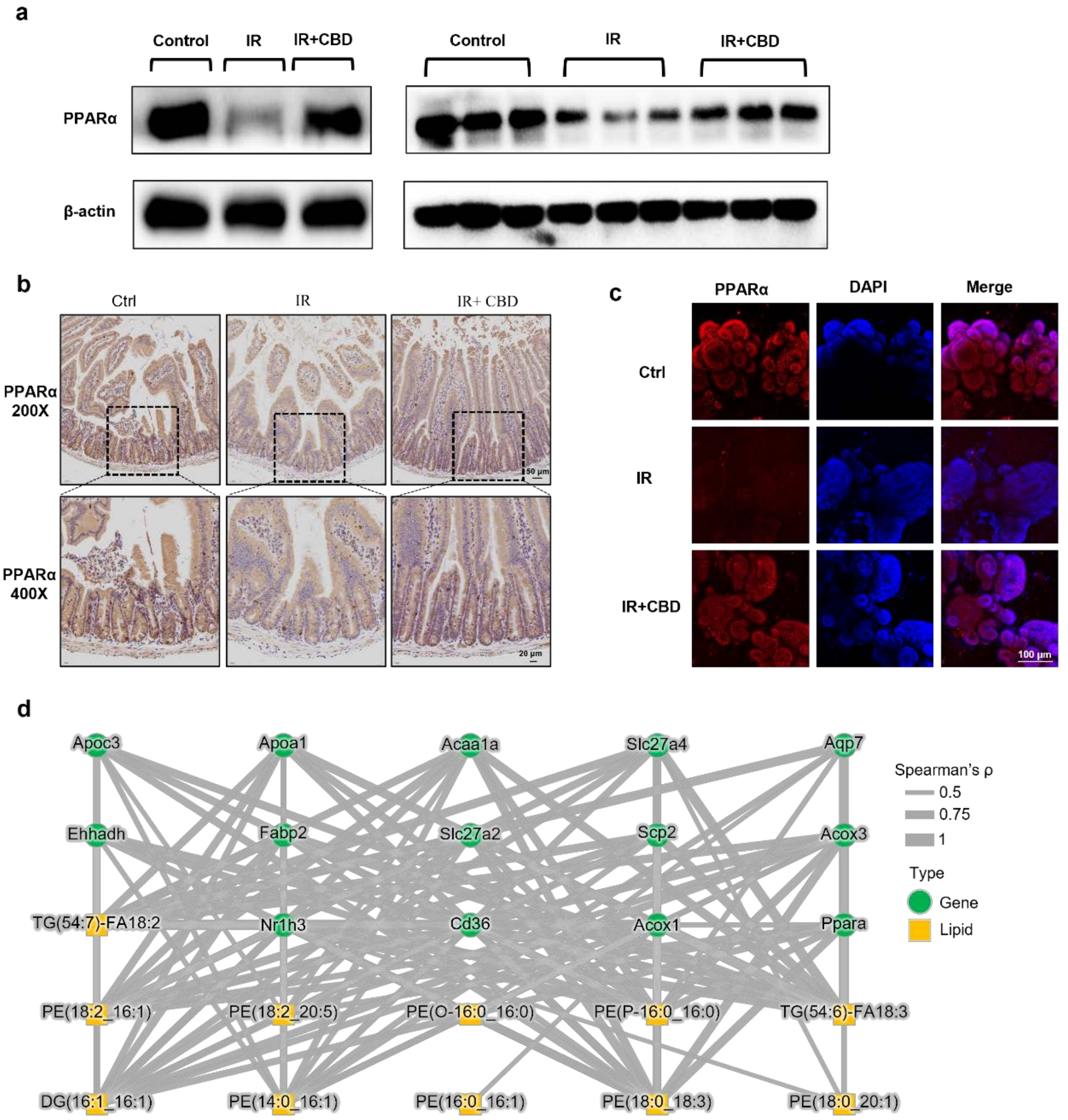

Supplementary Fig. 7.

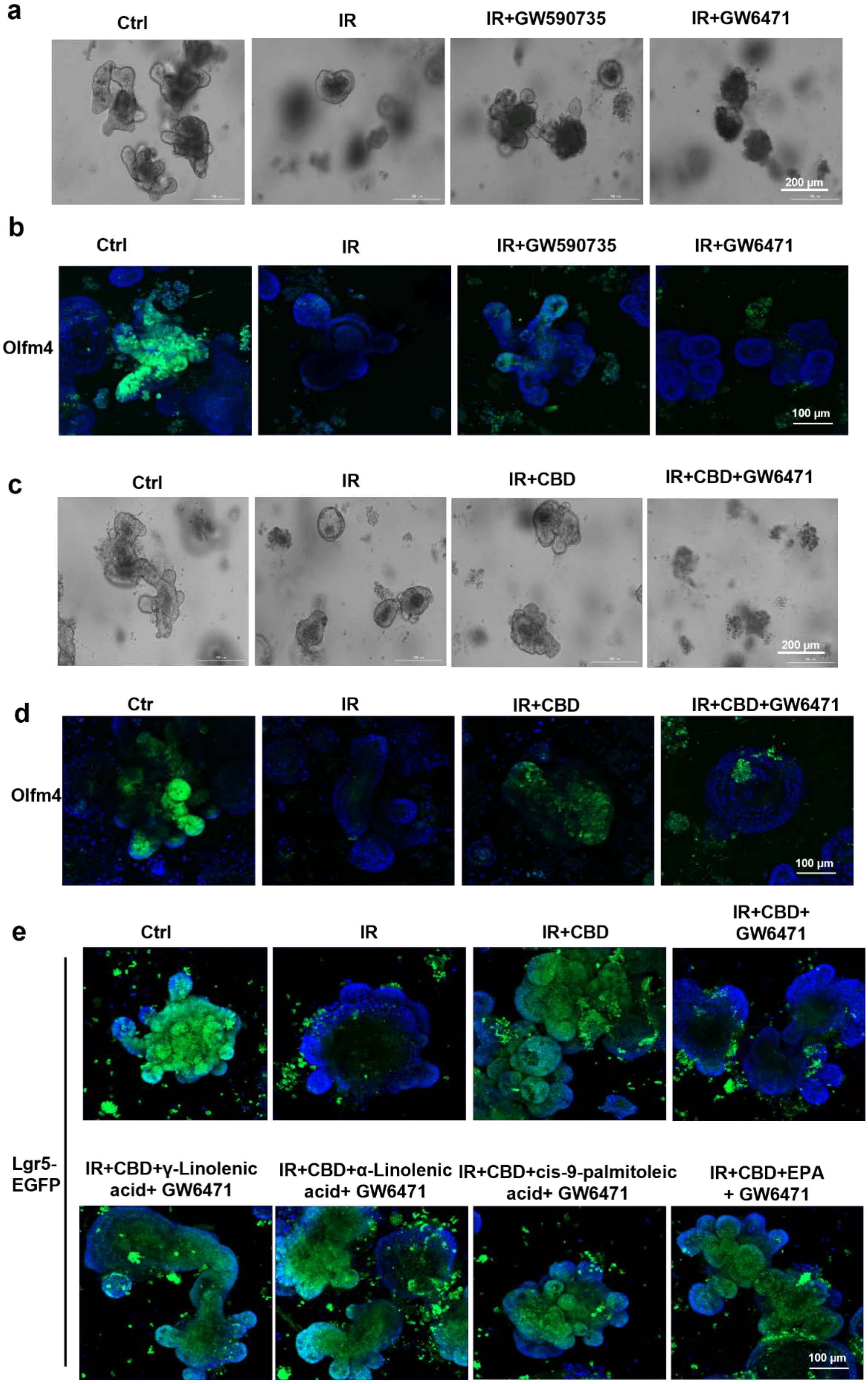

Supplementary Fig. 8.

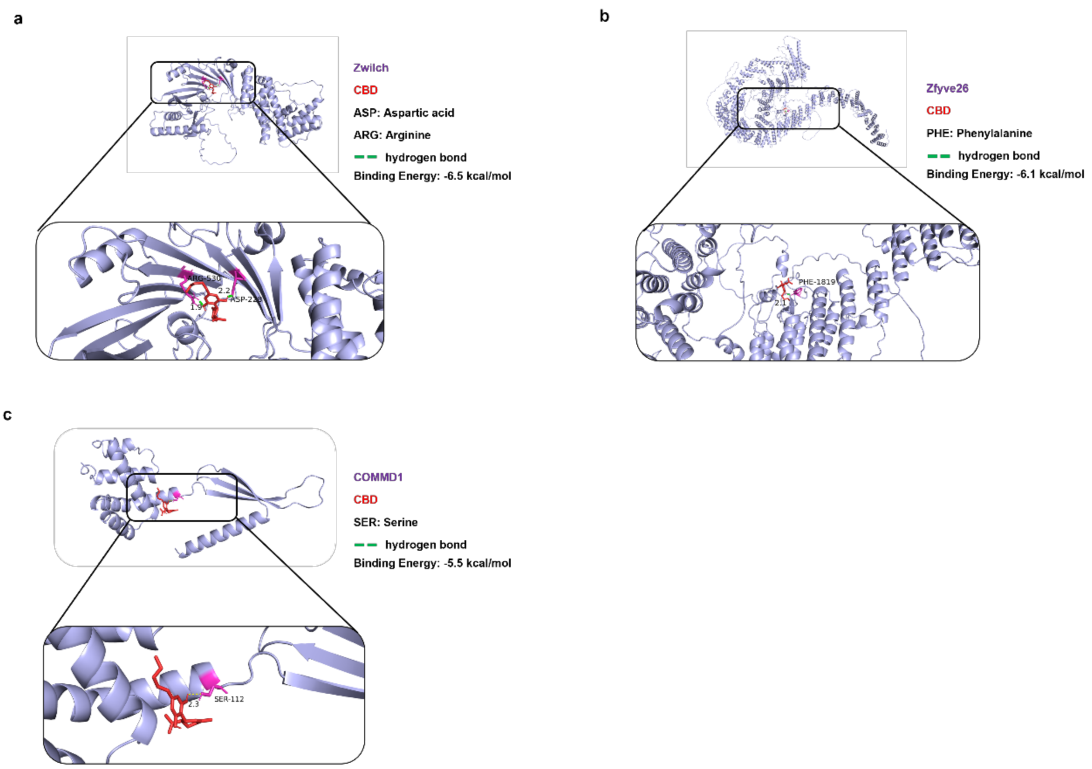

Supplementary Fig. 9.

Supplementary Tables

Supplementary Table 1. List of primers used for quantification of specific gene expression

Gene	Forward (5'–3')	Reverse (5'–3')
m-*Acaa1a*	TCTCCAGGACGTGAGGCTAAA	CGCTCAGAAATTGGGCGATG
m-*Acox1*	TAACTTCCTCACTCGAAGCCA	AGTTCCATGACCCATCTCTGTC
m-*Acox3*	ACCGGAAGAAAAAGACAGTGC	GAGGCTCTTGCTCGGTAGG
m-*Ehhadh*	ATGGCTGAGTATCTGAGGCTG	GGTCCAAACTAGCTTTCTGGAG
m-*Scp2*	CCTTCTGTCGCTTTGAAATCTCC	GCTTCCTTTGCCATATCAGGAT
m-*Slc27a2*	TCCTCCAAGATGTGCGGTACT	TAGGTGAGCGTCTCGTCTCG
m-*Slc27a4*	ACTGTTCTCCAAGCTAGTGCT	GATGAAGACCCGGATGAAACG
m-*CD36*	ATGGGCTGTGATCGGAACTG	GTCTTCCCAATAAGCATGTCTCC
m-*Ppara*	AGAGCCCCATCTGTCCTCTC	ACTGGTAGTCTGCAAAACCAAA
m-*Stat2*	TCCTGCCAATGGACGTTCG	GTCCCACTGGTTCAGTTGGT
m-*THOC3*	AGACACACCGCTCCAAAGC	GGACTGCACAGGCTTCAATTC
m-*GAPDH*	AAATGGTGAAGGTCGGTGTGAAC	CAACAATCTCCACTTTGCCACTG

Supplementary Table 2. The sequences of ShRNA in lentivirus

Oligonucleotides	Sequences
Non-targeting control	TTCTCCGAACGTGTCACGTAA
RNAi target of *Stat2*	GGCCAGAGACAGGGCTTAATT
RNAi target of *Thoc3*	GACGACGTTGTGACTTTCATT

Supplementary Table 3. List of primers of promoter region used for ChIP assays

Gene	Forward (5'–3')	Reverse (5'–3')
Slc27a2 (1166-1172)	AAGAGGTCAGAAGGCTATGAGAG	TGTGAGATATGAAAGAGGAACCAGT
Acox1 (1202-1208)	GCTGCCTTCAGTCCGATTCTATA	CTTCAAGTGGAGGTCTGAGGAT
Pparα (486-495)	GCAAGCACCTTTACCCATTCAA	CACCCACAGAACATTCCCTACT

Supplementary Table 4. List of primers for constructs in dual-luciferase assays

Gene	Sequence
h-Ppara-promoter-ZV702 (Forward)	CGATAGGTACCGAGCTCTTACGCGTGCTAGCCCAA ATGTCTGGTAGAACTGGGTTAACAGCAGAACACT
h-Ppara-promoter-ZV702 (Reverse)	TGCCAAGCTTACTTAGATCGCAGATCTCGAGCTCGC GCGTGCCCTCCAGCCCCTGGGCACCTGAGGCTGCA
h-Slc27a2-promoter-ZV702 (Forward)	CGATAGGTACCGAGCTCTTACGCGTGCTAGCATCCT GCATATCAGATATTAACATTCTGATTCATAA

h-Slc27a2-promoter-ZV702 (Reverse)	TGCCAAGCTTACTTAGATCGCAGATCTCGAGCCTAGGTAGTGGTACTGTACAAGGTTA
h-Acox1-promoter-ZV702 (Forward)	CGATAGGTACCGAGCTCTTACGCGTGCTAGCCTTATGCAGCTATAATTAAAACTCATGGTTCAAAGTGCT
h-Acox1-promoter-ZV702 (Reverse)	TGCCAAGCTTACTTAGATCGCAGATCTCGAGCTCACCTCCTTTCAGGCTAGGGCGGTGGGTGTGTGTGTGTG
h-Ppara-transcription factor (Forward)	GATTCTAGAGCTAGCATGGTGGACACGGAA
h-Ppara-transcription factor (Reverse)	TACAGGGACATGTACTGAAAGGATCTGCGATCGCTC
h-Stat2-transcription factor (Forward)	AGCTAGCGAATTCGGAGCCACCATGGCGCAGTGGGAAA
h-Stat2-transcription factor (Reverse)	ATGGACCCTTGATGCCTTCTGACTTCCTCGAGGGAGGTGGAGGTTCA
